# Comparative Dynamics of NMDA- and AMPA-Glutamate Receptor N-Terminal Domains

**DOI:** 10.1016/j.str.2012.08.012

**Published:** 2012-11-07

**Authors:** Anindita Dutta, Indira H. Shrivastava, Madhav Sukumaran, Ingo H. Greger, Ivet Bahar

**Affiliations:** 1 Department of Computational and Systems Biology, School of Medicine, University of Pittsburgh, Pittsburgh, PA 15213, USA; 2 Neurobiology Division, MRC Laboratory of Molecular Biology, Cambridge, CB20QH, UK

## Abstract

Ionotropic glutamate receptors (iGluRs) harbor two extracellular domains: the membrane-proximal ligand-binding domain (LBD) and the distal N-terminal domain (NTD). These are involved in signal sensing: the LBD binds L-glutamate, which activates the receptor channel. Ligand binding to the NTD modulates channel function in the NMDA receptor subfamily of iGluRs, which has not been observed for the AMPAR subfamily to date. Structural data suggest that AMPAR NTDs are packed into tight dimers and have lost their signaling potential. Here, we assess NTD dynamics from both subfamilies, using a variety of computational tools. We describe the conformational motions that underly NMDAR NTD allosteric signaling. Unexpectedly, AMPAR NTDs are capable of undergoing similar dynamics; although dimerization imposes restrictions, the two subfamilies sample similar, interconvertible conformational subspaces. Finally, we solve the crystal structure of AMPAR GluA4 NTD, and combined with molecular dynamics simulations, we characterize regions pivotal for an as-yet-unexplored dynamic spectrum of AMPAR NTDs.

## Highlights

► Crystal structure newly resolved for AMPAR GluA4 N-terminal domain (NTD) ► Dynamics underlying NMDA NR2B NTD allostery ► Similar global dynamics between AMPA- and NMDA-receptor NTDs ► Critical role of dimer interface contacts for GluA3 NTD allostery

## Teaser

Dutta et al. examine the dynamics and allostery of ionotropic glutamate receptors N-terminal domains (NTDs). Systematic comparison of the members of the AMPAR subfamily, and across the AMPAR and NMDAR subfamilies, reveals mechanisms of global and local motions favored by the NTDs and allosteric potential in AMPARs.

## Introduction

Ionotropic glutamate receptors (iGluRs) encompass a family of tetrameric glutamate-gated cation channels that mediate the majority of excitatory neurotransmission in the vertebrate central nervous system. Their operation underlies higher-order cognitive functions (Traynelis et al., 2010). Aberrant iGluR signaling is associated with several acute and chronic neurodegenerative diseases. The iGluR family harbors three major subfamilies: α-amino-3-hydroxy-5-methyl-4-isozazolepropionic acid receptors (AMPARs), kainate receptors (KRs), and *N*-methyl-D-aspartate receptors (NMDARs). Sequence similarity and structural data suggest a shared architecture between these subfamilies: an extracellular domain (ECD), a transmembrane domain (TMD), and an intracellular carboxyl-terminal domain that mediates trafficking and anchorage at synaptic sites (Traynelis et al., 2010; Dingledine, 2010). The ECD consists of a distal N-terminal domain (NTD) and ligand-binding domain (LBD).

**Figure 1:**
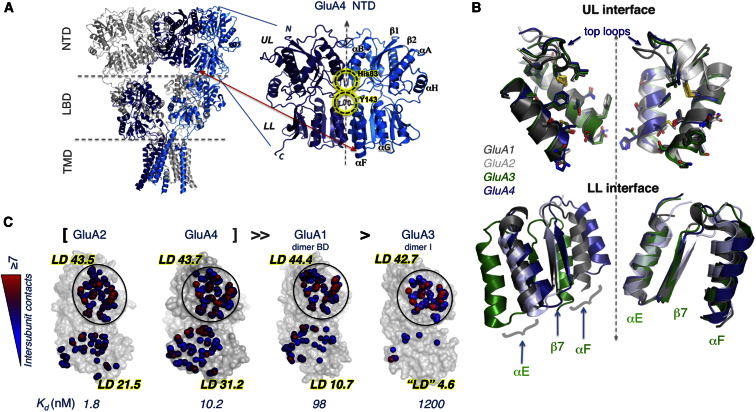
Structure of the GluA4 NTD Facilitates a Comparative Structural Analysis (A) Intact structure of GluA2 AMPAR (left) displaying the spatial arrangements of four subunits (two shown in gray and the others in blue and dark blue) that span the three domains (NTD, LBD, and TMD). The location of the NTD dimer resolved for GluA4 is enlarged. Subunits are symmetrically positioned, each consisting of an upper lobe (UL) and a lower lobe (LL); secondary structural features (helices αA, αB, αE, αF, αG, and αH and strands β1 and β2) are labeled. Interfacial interactions are highlighted. (B) UL dimerization interfaces of GluA1–A4 are largely conserved but LL packing shows heterogeneity. UL interfaces of GluA1–GluA2 (grays), GluA3 (green), and GluA4 (blue) have been artificially separated to show the structural conservation and orientations of key residues (shown in stick) making contacts across the interface. Two-fold axis of symmetry is shown as a dashed line. Superposition of LL shows distinct differences in interface packing that is most prominent in GluA3. (C) Intersubunit contacts at the UL and LL interfaces of GluA1–A4 NTDs. Atoms making interfacial contacts within 4.5Å are shown as spheres and colored from blue (one contact) to red (≥7 contacts). Calculated local contact density (LD) indices and empirically measured dimer dissociation constants (*K*_*d*_) are also shown. The four NTDs are ranked by their homodimerization affinity. Note the LL interface is highly variable between AMPAR paralogs, whereas the UL interface is largely invariant. See also Figure S1 and Tables 1 and S1.

The full-length structure of the GluA2 AMPAR (Figure 1A) provided a first view of the modular arrangement of the individual subunits of iGluRs (Sobolevsky et al., 2009). Both the NTDs and LBDs feature the clamshell-like bilobate fold belonging to the periplasmic binding protein (PBP)-like family (Quiocho and Ledvina, 1996). The function of the LBD is well characterized (Armstrong and Gouaux, 2000); L-glutamate docking to the cleft between the upper lobe (UL) and lower lobe (LL) of each subunit results in cleft closure, which is allosterically transmitted to the TMD to trigger channel activation. The function of the NTD remains to be established. In NMDARs, the NTD contributes to signaling as a key allosteric modulator of channel open probability (Gielen et al., 2009). However, the mechanism of NMDAR NTD allostery is unclear as currently available crystal structures with and without the interlobe Zn^2+^ ligand look almost identical (Karakas et al., 2009). NTD allosteric activity in non-NMDARs (i.e., AMPAR and KRs) remains poorly understood and is a matter of debate.

iGluR NTDs are organized in the tetrameric structure as dimeric entities, structurally homologous to metabotropic glutamate receptors (mGluRs) (Kunishima et al., 2000; Muto et al., 2007), and natriuretic peptide receptor (NPR) ligand-binding cores (He et al., 2006), which also operate as dimers. Recent structural data underscore a mechanistic basis for allosteric signaling via NMDAR NTDs, where the LLs are separated and thus free to move in response to ligand binding (Farina et al., 2011; Karakas et al., 2011). This arrangement mimicks the ligand binding cores of mGluRs and NPRs but is different from AMPAR and KR NTD dimers that are mostly “zipped-up” across both lobes providing an extensive assembly interface (Kumar et al., 2009; Clayton et al., 2009; Jin et al., 2009; Rossmann et al., 2011), which likely restricts lobe motions. Accordingly, AMPA- and NMDA-R NTDs would have evolved different functions, with the AMPAR NTD mainly directing subunit assembly (Ayalon and Stern-Bach, 2001; Hansen et al., 2010; Rossmann et al., 2011) but with the NMDAR NTD additionally modulating ion channel function. This latter role has led to a surge in NMDAR modulator development, some of which have entered clinical trials (Mony et al., 2009; Karakas et al., 2011). Utilizing a combination of X-ray structural approaches and normal mode analysis (NMA) with elastic network models (ENMs), we showed recently that the NTDs of AMPARs do have access to well-defined collective fluctuations (Sukumaran et al., 2011). Together with biophysical measurements of NTD intrinsic fluctuations (Jensen et al., 2011), these recent results exemplify the dynamic capability of non-NMDAR NTDs and suggest a potential allosteric signaling capacity, which would provide a currently unexplored target for channel modulators.

To better understand this discrepancy between iGluR NTD activities, we set out to describe and compare NTD motions between the AMPAR and NMDAR subfamilies at multiple levels. We first solved the crystal structure of the remaining AMPAR NTD, GluA4, and conducted a comprehensive analysis of NTD dynamics across the entire subfamily of AMPARs using both NMA with anisotropic network model (ANM) and full-atomic molecular dynamics (MD) simulations; we also characterized the currently elusive dynamics of the NMDAR NTD, a powerful allosteric modulator. Combined with MD and ANM results, we determine the mechanisms of global and local motions favored by the iGluR NTD architecture, identify critical residues facilitating these motions, and reveal a mechanistic link between LL interface stability and cleft movements, which vary among subfamily members. Finally, upon comparison, we find that cooperative modes of motion intrinsically accessible to AMPAR NTD monomers are almost identical to those of NMDARs, albeit spatially more restricted upon dimerization. Significantly, AMPAR NTDs possess the ability to readily reconfigure into NMDAR NTD conformers. Together, these data reveal an unexpected parallel between AMPAR and NMDAR NTDs and provide a glimpse into the dynamic landscape of iGluR NTDs.

## Results

### The GluA4 NTD at 2.2 Å

The NTD encompasses the most sequence-diverse part of the receptor, with the four AMPAR paralogs only sharing ∼55% sequence similarity. In addition, the packing between NTD dimers is diverse (Rossmann et al., 2011). Hence, we first solved the structure of the remaining GluA4 NTD to facilitate a comprehensive analysis of this domain across the AMPAR subfamily (Figure 1A). GluA4 NTD crystals diffracted to 2.25 Å (Table 1); the asymmetric unit contained one protomer exhibiting the conserved bilobate PBP-fold seen in all AMPAR and KR NTDs crystallized to date (Mayer, 2011), with its dimeric partner readily observed by crystal symmetry. Overall, GluA4 resembles other AMPAR (GluA1–GluA3) NTDs with root-mean-square deviation (rmsd) values of ∼1.2 Å when superimposing individual NTD protomers and up to 4.1 Å when superimposing the NTD dimers (see Figure S1A for monomers; Table S1 for dimers [available online]). As in GluA1 and GluA3 (Sukumaran et al., 2011; Yao et al., 2011), no ligand density was evident in the interlobe binding cleft, and lobe opening angles were similar between GluA1–A4.

The UL interface is the most highly conserved region between AMPAR NTD paralogs (Figure S1B). Packing along the GluA4 upper lobe (UL) dimer interface is indeed comparable to that of other AMPAR NTD subtypes, with variations mostly in positioning of the top/selectivity loops (Figures 1B and S1C). An interesting difference is His83 projecting from the base of α helix C (αC) across the GluA4 dimer interface; this residue is replaced by Asn in GluA1–A3 (Figures 1B and S1D). The LL interface is more variable in primary sequence and in packing geometry between GluA1–A4 (Figure 1B). Contacts across the LL interface of GluA4 are comparable to GluA2 (Protein Databank Bank [PDB] ID code 3HSY), to GluA3 dimer *BD* (PDB ID code 3P3W; italicized letters indicate chain identifiers from PDB structures, i.e., protomers *B* and *D* from ID code 3P3W), and to GluA1 dimer *AC* (PDB ID code 3SAJ) (Yao et al., 2011). However, in GluA3 dimers *CD* (PDB ID code 3O21) and *AC* (PDB ID code 3P3W), the inter-LL distance is wider (Sukumaran et al., 2011) (Figure 1B). Also in GluA1 (dimer *BD*; PDB ID code 3SAJ), the LLs are packed more loosely partly because of rotations of Leu137 away from the dimer interface. At the equivalent position, GluA4-selective Tyr143 side chains stack across the interface, separated by ∼3 Å (Figure S1D). Thus, UL interface contacts are mostly conserved in AMPAR NTDs, whereas LL packing is diverse and subunit selective. The looser LL contacts in GluA1 and GluA3 correlate with reduced NTD dimer stabilities (Rossmann et al., 2011).

To quantify contacts across the structurally variable LL interfaces, we calculated local atomic contact density (LD) indices, a measure for packing density across interfaces (Bahadur et al., 2004; Sukumaran et al., 2011) (Figure 1C). GluA4 exhibits interface characteristics similar to those of GluA2 (UL interface LD: 43.7, LL interface LD: 37.2). GluA1 shows high contact density in the UL (LDs of 40.8 and 44.4 in both dimers *AC* and *BD*), characteristic of tight, biologically relevant interfaces (Bahadur et al., 2004). However, the LL interface shows variability: GluA1 dimer *AC* is similar to those of GluA2 and GluA4, whereas *BD* is similar to GluA3 (*CD*; PDB ID code 3O21). Again, these structural features agree with measurements of AMPAR NTD homodimer stabilities by analytical ultracentrifugation (Rossmann et al., 2011), where GluA3 exhibited the weakest affinity (K_d_ ∼1 μM), followed by the intermediate GluA1 (K_d_ ∼100 nM), likely reflecting their LL separations and multiplicity of dimeric forms in crystal structures. GluA2 and GluA4 featured K_d_ values between 2 and 10 nM, consistent with tighter LL packing (Zhao et al., 2012). Thus, the greatest structural variability between AMPAR NTDs is at the LL dimer interface; looser LL packing is expected to increase NTD interprotomer mobility.

### Comparative ANM Analysis Reveals Global Motions Shared by AMPAR NTDs

To compare the structural dynamics between AMPAR NTDs and to contrast those to the allosterically active NMDAR NTDs, we first analyzed the collective motions of GluA1–A4 dimers using the ANM (Atilgan et al., 2001; Eyal et al., 2006). ANM is particularly suitable for a comparative assessment of global motions intrinsic to whole protein families (Bahar et al., 2010b). Global motions are those at the lowest frequency end of the mode spectrum, predicted by the ANM to be uniquely defined by the native fold. The lowest frequency mode, *mode 1*, represents a structural change (usually subunit/domain movements) along the softest/smallest ascent direction away from the original energy minimum (Bahar et al., 2010a).

**Figure 2:**
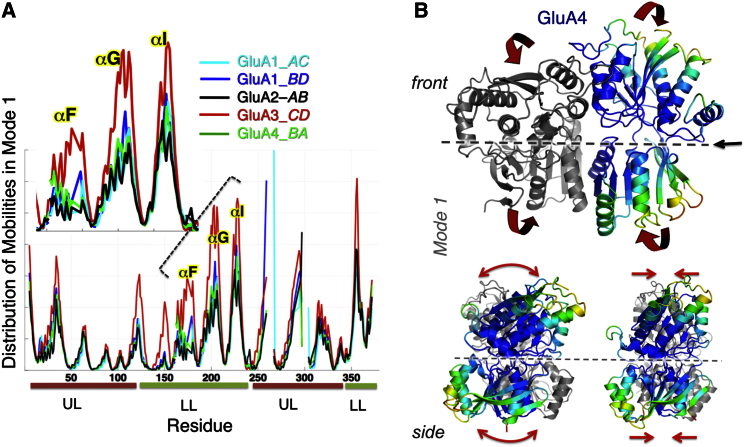
Global Dynamics of GluA4 Dimer in Comparison to other AMPAR NTDs Probed by ANM (A) Distribution of square displacements of residues in the most global (lowest frequency) mode intrinsically accessible to AMPAR NTD dimers (GluA1-*AC*, GluA1-*BD* [3SAJ], GluA2-*AB* [3HSY], GluA3-*CD* [3O21], and GluA4 [PDB ID code 4GPA]). The four subtypes show similar profile (see the high correlations listed in Table S2) but different size motions (see Table S3). (B) Shared mechanism of global motion: counterrotation of the two protomers (indicated by red arrows), depicted for GluA4 as a representative structure, from the front and side views. The diagram is color-coded from red (most mobile in mode 1) to blue (least mobile). The global mobility rank of the four AMPAR NTD dimers is GluA3-*CD* (0.110) > GluA1-*BD* (0.169) > GluA1-*AC* (0.184) ≈ GluA4-*BA* (0.187) > GluA2-*AB* (0.187). The numbers in parentheses indicate the global mode eigenvalues (see Experimental Procedures). See also Table S1.

ANM calculations performed for GluA1–A4 NTD dimers showed that the AMPAR subfamily members share a common mechanism of global reconfiguration along mode 1 with a correlation of 0.90 ± 0.04 (Figure 2; Table S2A): torsional counterrotation of the two protomers, as previously noted for GluA2 and GluA3 (Sukumaran et al., 2011) (Movie S1), and extended here to the entire subfamily (Figures 2A and 2B). The four subunits exhibit similar global mode shapes (distribution of mobilities, Figure 2A); their motion amplitudes (peak heights), however, show variations, with GluA3 exhibiting higher mobilities (Table S3), particularly in the LL (residues 120–225; inset of Figure 2A). These data, that is, the flexibility between paralogs (Figure 2A, inset), directly correlate with experimental measurements of AMPAR NTD dimer K_d_'s (Figure 1C) (Rossmann et al., 2011).

### Intrinsic Ability of NMDAR to Sample Open and Closed Cleft Conformations Supported by ANM and MD

NMDAR NTDs allosterically modulate NMDAR ion channel function, triggered by small molecule ligands and Zn^2+^ ions that bind the dimer interface and cleft region between lobes, respectively. However, current NMDAR NTD structures are similar with regard to cleft-opening angle (Karakas et al., 2009) and protomer conformation upon ligand binding (Karakas et al., 2011). Thus, the motions underlying NMDAR NTD allostery are unknown.

**Figure 3:**
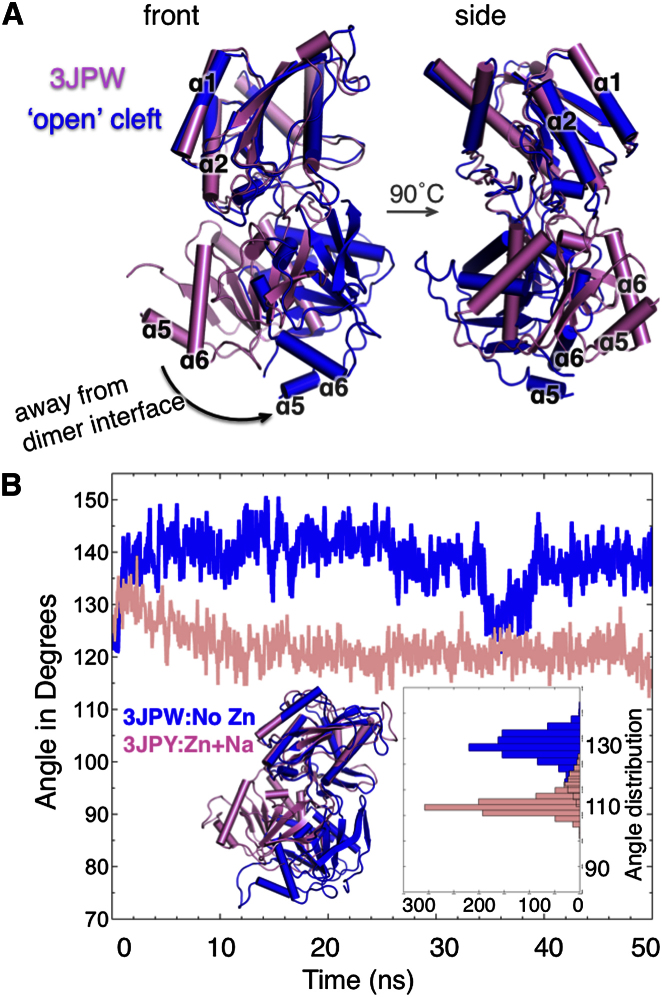
Intrinsic Ability of NMDAR NTD to Undergo Cleft Motions (A) Deformation of NR2B subunit (PDB ID code 3JPW, pink) along ANM mode 2, leads to opening of the cleft (blue). (B) The time evolution of the cleft angle observed in the MD runs NMDA1 (pink, in presence of Zn^2+^) and NMDA2 (blue, in absence of Zn^2+^). The cartoon in the inset is the superposition of 50 ns snapshots from NMDA1 and NMDA2. It illustrates the opening of the cleft in the simulation performed without Zn^2+^ similar to the global reconfiguration predicted by the ANM for 3JPW in (A). The histograms in the inset are of the distribution of the angles sampled by NR2B in the two simulations: the average angle is 138° in NMDA1 and 121° in NMDA2. See also Table S4.

Our ANM analysis of the global dynamics of the NMDAR NTD, performed for the NR2B subunit, revealed a global twist of the LLs toward the dimer interface accompanied by cleft opening, whereas LL twist motion in the opposite direction induced cleft closure, highlighting the classic clamshell-like motion (Figure 3A; Movie S5). Full-atom molecular dynamic (MD) simulations (Table S4) performed for the same subunit in the apo (NMDA1) and Zn^2+^-bound (NMDA2) states also revealed an overall rigidification accompanied by cleft-angle closure in the presence of Zn^2+^ (Figure 3B, pink curve), whereas cleft opening was observed (blue curve) in the absence of Zn^2+^. The cleft angle was monitored based on the relative positions of L124 (UL), S149 (cleft), and I257 (LL) α-carbons. The apo form thus stabilizes a more open conformation by at least 17° compared to the Zn^2+^-bound form (Figure 3B, inset). Strikingly, the same type of structural change is predicted by the ANM mode 2 for NR2B (Figure 3A). Thus, both ANM and MD support a classic periplasmic-binding protein mode of ligand recognition for Zn^2+^ binding.

### Similarity between the Intrinsic Dynamics of NMDAR and AMPAR NTD Protomers

**Figure 4:**
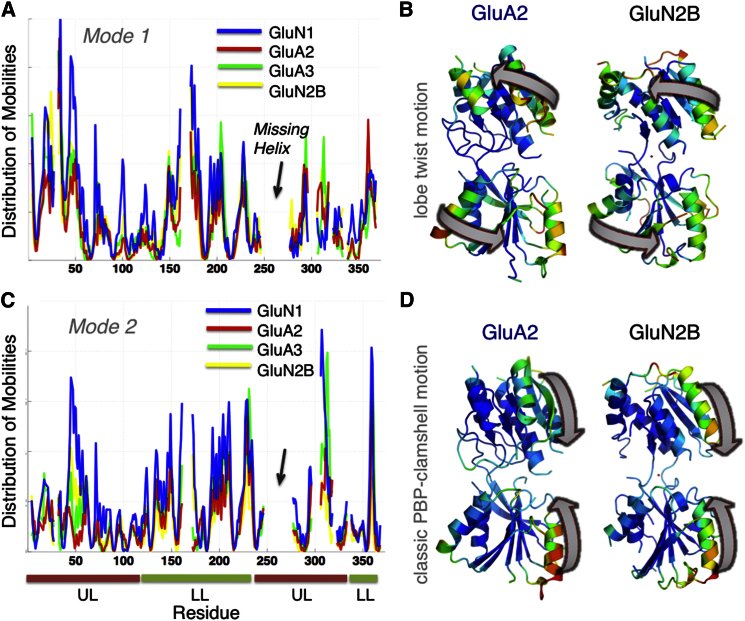
Comparing the Global Dynamics of NTD Protomers Resolved for AMPA and NMDA Receptors (A) Comparison of the mobility profiles as driven by the lowest frequency (most cooperative) mode of motion accessible to GluN2B (3JPW), GluN1 (3Q41-*A*), GluA2 (3HSY-*B*), and GluA3 (3O21-*C*) NTD monomers. The abscissa in (A) is labeled according to residues in GluA2. (B) Ribbon diagram of a representative AMPAR (GluA2) and an NMDAR (GluN2B) NTD monomer, colored by the mobility profile in mode 1. The arrows indicate the mechanism of motion (counterrotation of the two lobes). (C and D) Same as in (A) and (B), for ANM mode 2, a clamshell-like opening/closing of the two lobes. See also Tables S2 and S3.

Next, we compared global dynamics between AMPAR and NMDAR NTD monomers. Despite their distinctive structural features (Furukawa, 2012), the global modes between the two subfamilies are surprisingly preserved. Figure 4 illustrates the results for AMPAR GluA2 and GluA3 and NMDAR GluN1 and GluN2B subunits. Two dominant modes of motion are observed: counterrotation between the two lobes (mode 1, panels A and B; Movies S2 and S3) and intralobe clamshell opening/closing (mode 2, panels C and D; Movies S4–S5). The global modes of all AMPAR and NMDAR NTD monomers exhibited a high level of similarity, with correlation coefficients varying in the range 0.73 ± 0.11, highlighting the *universality* of the observed motions despite stark differences in tertiary and quaternary packing (Table S2B). Especially, the clamshell-like motion seen in NR2B (Figure 3), which enables sampling of ligand unbound/bound conformations, is also preserved in AMPAR monomers.

Although global mode shapes are similar between GluN1, GluN2B, GluA2, and GluA3 monomers (Figures 4A–4C), the relative amplitudes are largest in GluN1 and smallest in GluA2. Table S3 shows an overall ranking of GluN1 > GluA3 ∼GluN2B > GluA1 > GluA4 ∼GluA2 (stiffest) based on mode 1 (see Experimental Procedures), and a similar trend is observed in mode 2. This analysis reveals an unexpected difference between GluN1 and GluN2B. Importantly, these modes of motions and intrinsic flexibility are largely conserved between NMDA- and AMPAR-NTDs, as discussed further below.

### Effect of Dimeric Packing on the Intrinsic Dynamics of AMPAR and NMDAR NTD Monomers

Because AMPAR- but not NMDAR-NTDs assemble into stable homodimers (Clayton et al., 2009; Jin et al., 2009; Rossmann et al., 2011; Zhao et al., 2012), we next evaluated the changes in dynamics upon NTD dimerization, using a perturbation method (Zheng and Brooks, 2005; Ming and Wall, 2005b), which facilitates assessing the effect of environment on the dynamics of examined systems. Here, each monomer in the dimeric NTD of AMPAR (GluA2 and GluA3 homodimers) and NMDAR (N1-N2B heterodimer) is taken as the *system* and is analyzed in the context of the other monomer, which represents its *environment*. The dynamics in the presence of the environment is then compared to that of the system in isolation (i.e., the intrinsic dynamics of the monomers presented above).

**Figure 5:**
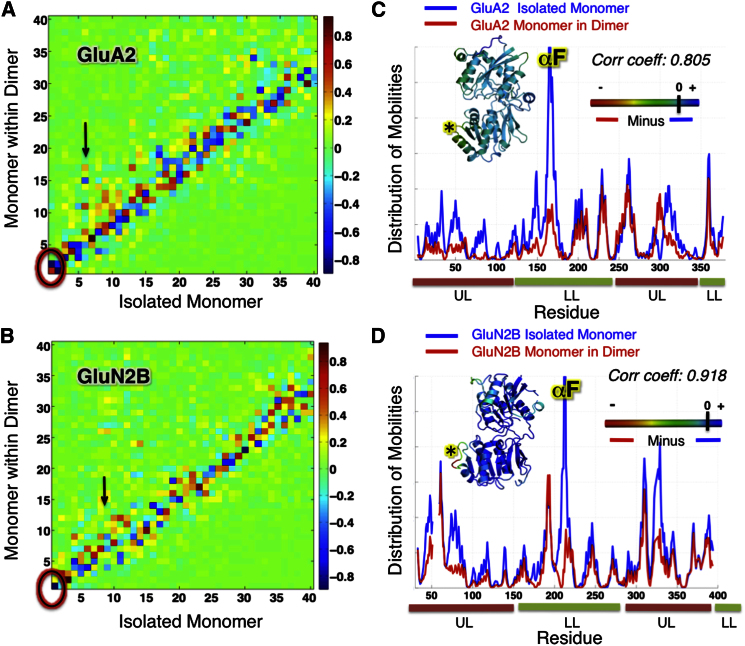
Effect of Dimerization on the Intrinsic Dynamics of AMPAR and NMDAR NTD Monomers (A) Correlation between top 40 modes accessible to GluA2 protomer in isolation (3HSY-*B*; *abscissa*) and in the dimer (3HSY; *ordinate*). Darkest red and blue regions refer to strongest correlations (see the scale on the right). Clamshell motions (monomer mode 1) are maintained in the dimer but manifested by mode 2 (circled region). (B) Same as (A), for GluN2B (3QEL-*D*) monomer compared to GluN1/GluN2B heterodimer (3QEL). (C and D) Mobility profiles for GluA2 and GluN2B monomers in isolation and in the dimer, showing the suppression of mobilities (at the UL in particular) upon dimerization (see Figure S2 for GluA3 and GluN1). Insets show GluA2 and GluN2B monomers colored by their change in mobility upon dimerization, from most suppressed (red) to unaffected (blue).

The maps in Figures 5A and 5B display the correlations between the top-ranking 40 ANM modes predicted for the isolated and dimeric forms of the monomers of GluA2 and GluN2B. Highest correlations are indicated by correlation cosines (see Experimental Procedures) close to ±1 (colored red/blue) and lack of correlation by values approaching zero (green). The observed high correlations along the diagonal indicate that the dynamic character of the monomer is maintained in the dimer, with minor alterations (and occasional swaps in the order of mode).

Although the shapes of the global modes are closely maintained, the amplitudes of the motions exhibit a dependence on dimerization. One would expect the amplitudes of fluctuations to be depressed by interprotomer interactions, especially at interface regions. This is the case for GluA2, GluA3, and GluN2B (Figures 5C and 5D; Figure S2C), where the protomer in the dimer exhibits lower mobility compared to the isolated monomer. The insets in Figures 5C and 5D show the ribbon diagrams of the GluA2 and GluN2B monomers, respectively, colored by the difference in mobility between the monomer in the dimeric system and the isolated monomer. The region that shows the largest suppression is αF in the LL followed by the UL interface, whereas UL and LL cores remain unchanged. Dimerization has almost no effect on the mobility of GluN1, that is, interprotomer interactions do not obstruct the conformational flexibility of this NTD (Figure S2D; Table S3). Notably, the suppression of mobility in the αF helix region may have implications on the allosteric capacity of AMPAR NTDs.

### NMDAR and AMPAR NTDs Readily Reconfigure along a Single, Global Mode of Motion

The observed difference in the size of global motions between GluN1 and AMPAR NTD protomers are likely due to their differences in dimeric packing. We next determined whether dimer conformations are interconvertible between iGluR subfamilies. If high-energy barriers separate different dimeric forms and preclude structural rearrangement, their distinctive (nonconvertible) interprotomer packing would impact their dynamics, and the known allosteric capacity of the NMDAR NTDs could be attributed to higher conformational freedom. If, however, the different structures are alternative forms readily accessible via soft modes of motions, this would imply that the seemingly less mobile AMPAR NTDs (such as GluA2 and GluA4) can access conformers with known allosteric potential (i.e., NR2B).

**Figure 6:**
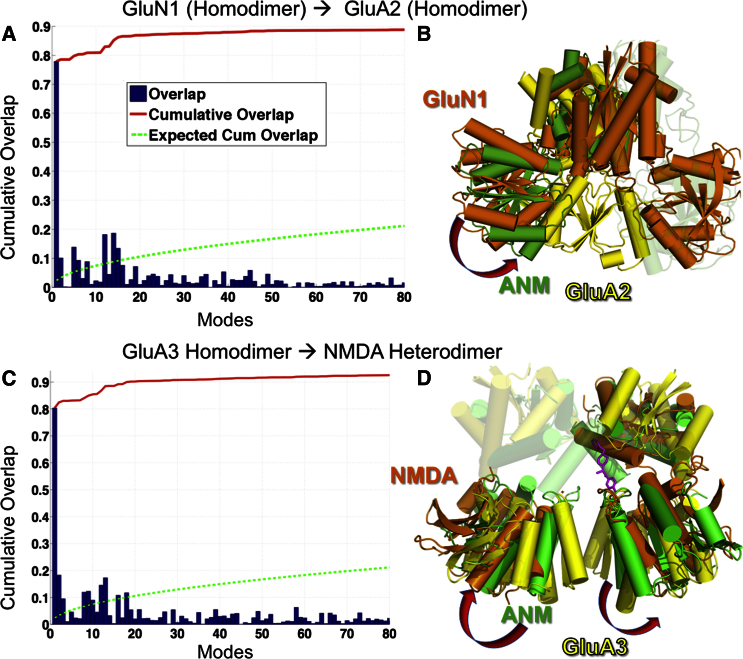
Ease of Transition between Dimeric Conformers of NMDAR and AMPAR NTDs (A) Results are illustrated for the passage from GluN1 (NMDA) homodimeric conformer to GluA2 dimer conformer. The overlap (blue bars) represents the correlation cosine (see Experimental Procedures) for each of the top-ranking 80 ANM modes to the conformational change. The red curve represents the cumulative overlap, adding up the contribution of all modes starting from the low frequency end (mode 1). The dashed green curve displays the *control*, for random modes. The slowest mode predicted for GluN1 (PDB ID code 3Q41-*AB*) yields an overlap of ∼80%, indicating a strong predisposition of the GluN1 homodimer to assume the conformation of the GluA2 dimer. (B) Two transitional end points (orange, yellow) and an intermediate structure reached by moving exclusively along mode 1 (green). (C and D) Same as (A) and (B), for the change in the conformation of GluA3 homodimer (yellow) toward that of the heterodimer GluN1/GluN2B (PDB ID code 3QEL-*CD*, orange) along GluA3 ANM intermediate (green). See also Figure S3.

To make a quantitative assessment of the ease of transition between different NTD dimers, we examined the *overlap* (see Experimental Procedures) between (1) structural difference, Δ{R}_S1_→_S2_ = {R_*0*_}_S1_ − {R_*0*_}_S2_, that is required for the transition from dimeric conformer “S1” to conformer “S2” (based on PDB coordinates, after optimal superimposition of the endpoints), and (3) the soft modes of structural change favored by S1, as predicted by the ANM. A strikingly easy “conversion” between AMPAR and NMDAR NTD conformers is evidenced by the high overlap between Δ{R}_S1_→_S2_ and mode 1 predicted for S1. Figures 6A and 6B illustrate the results for GluN1→GluA2 and GluA3→GluN1-N2B, respectively. The former provides evidence for the ease of transition from NMDAR (GluN1) homodimer to the GluA2 homodimer and the latter from the GluA3 homodimer to the GluN1/GluN2B heterodimer (Figures 6C and 6D; see also Movies S6 and S7). This analysis underscores the significance of global modes in providing access to functional conformers. For example, upon deforming GluA3 NTD along ANM mode 1 alone, the rmsd from the GluN1-N2B heterodimer decreases from 13.06 to 6.12 Å (more than 50%).

Results for other pairs of conformers between AMPAR and NMDA subfamilies are shown in Figure S3. Ninety percent cumulative overlap with the targeted deformation (red curve) is attainable with a small subset (e.g., 20–25) of soft modes (out of a total of ∼1,800 ANM modes) in all cases, except for the GluA2 → GluN1 homodimer, in which the overlap is ∼70%. Together with the ANM data (Figures 2, 3, 4, and 5), these results underscore an unexpected parallel between AMPAR and NMDAR NTD flexibility and dynamics.

### All-Atom MD Simulations Indicate High Intra- and Inter-LL Mobilities in AMPAR NTDs

To obtain a better understanding of the molecular interactions that underlie iGluR NTD dynamics, we conducted all-atom MD simulations. Root-mean-square fluctuations (rmsfs) in residue positions (Figure S4) confirm that GluA3 exhibits the highest mobility among all AMPAR NTDs. This enhanced mobility is primarily mediated by LL helices αE and αF, in agreement with data from fluorescence correlation spectroscopy experiments (Jensen et al., 2011). These helices may make contacts with the LBD in the intact structure of AMPAR (Figure 1A) and could thus communicate to downstream segments of the receptor. Similarly, helix αH located next to the entrance of the cleft in AMPAR NTDs features high mobility, consistent with the structural variation observed upon superposition of GluA1–A4 structures (Figure S1A).

**Figure 7:**
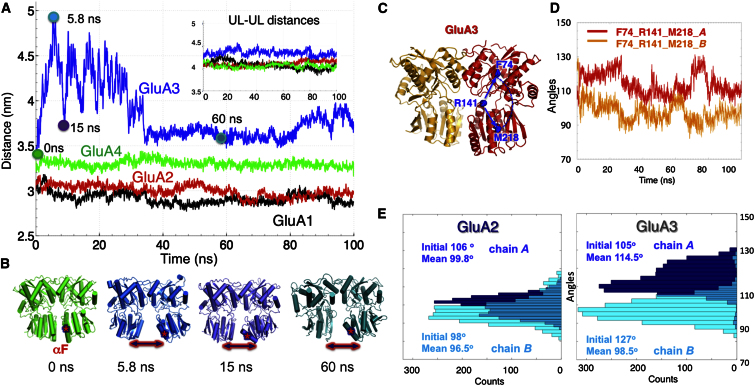
Lower Lobe Interface Instability of GluA3 Evidenced by Comparative Analysis of MD Simulations for GluA1–A4 (A) Distance between the mass centers of LLs, shown for GluA1–A4 NTDs as a function of simulation time. Results for the ULs are shown in the inset. Large fluctuations are observed in GluA3 LL-LL distance (blue trace). (B) Snapshots display GluA3 conformations at t = 0, 5.8, 15, and 60 ns (see colored circles in A). (C) Probe residues selected for monitoring the changes in interlobe cleft angle, shown for GluA3 NTD (3O21-*CD*). (D) Time evolution of interlobe angle for GluA3 protomers. Note the periodic opening/closing and the anticorrelation between the protomers. (E) These properties are contrasted to those observed for GluA2, where the angles exhibit minimal fluctuations. Histograms refer to interlobe angles for protomers *A* (dark blue) and *B* (cyan). See also Figures S4,S5 and,S7 and Table S4.

Next, we monitored interlobe (UL-UL and LL-LL) distances based on their centers of mass (CMs). Simulations clearly show that the UL-UL distances (∼4.0–4.3 nm) are maintained in all AMPAR NTD dimers (inset of Figure 7A), whereas LL-LL distances vary between dimers: they maintain their original values (of 2.9–3.3 nm) in GluA1, GluA2, and GluA4. In GluA3, however, they increase to more than 4.5 nm at early stages of the simulation, essentially disrupting the LL interface (Movie S8). Snapshots of the GluA3 NTD at different stages (Figure 7B) illustrate the loss of the LL interface within the first 5 ns, followed by stabilization of a different conformation distinguished by the loss of αE helicity and the reorientation of αF toward the LBD. As shown below, this behavior is due to the unique positive charge distribution in the GluA3 LL interface (Sukumaran et al., 2011). GluA3 also exhibits localized rearrangements in the UL dimer interface, which are not seen in the other AMPAR paralogs. Specifically, hydrophobic packing is disrupted as phenylalanine pairs (F56, F88) are separated and in some cases, irreversibly broken (Figure S5). This “acquired” UL instability further points to the unique behavior of the GluA3 NTD, potential coupling between the LL and UL interfaces in AMPAR NTDs, and the importance of the LL as a key structural determinant mediating intrinsic dynamics.

### GluA3 NTD Protomers Undergo Clamshell-like Motions

To determine the influence of interface stability on classic PBP-like clamshell motions (Quiocho and Ledvina, 1996; Trakhanov et al., 2005), we examined the fluctuations in the interlobe cleft angle, based on three C^α^-atoms in each AMPAR NTD (Figure 7C). We observe markedly larger angular fluctuations in GluA3 than in GluA2 (Figures 7D and 7E); GluA3 featured the widest opening of interlobe cleft angle (ranging up to >130° in protomer *A*). Interestingly, the two GluA3 protomers appear to undergo anticorrelated fluctuations, with protomer *A* closing and *B* opening with a periodicity of ∼25 ns (Figure 7D). This motion is unique to GluA3 and not discernable in other AMPAR counterparts, suggesting that LL flexibility in GluA3 may be coupled to clamshell-like motions of the individual protomers. Moreover, cleft motions in GluA3 (Figure 7D, orange curve) are accompanied by changes in UL hydrophobic packing (Figure S4), together suggesting a coupling between clamshell-like motions of the individual protomers and interprotomer packing.

### Effect of LL Residues on Interlobe Packing and Dynamics

The difference in GluA2 and GluA3 interface stability and residue fluctuations observed in MD simulations is also reflected in their dimer stabilities derived experimentally (Rossmann et al., 2011). In GluA2, hydrophobic residues contribute to the LL-LL contacts, whereas in GluA3 pairs of arginines (R163 and R184) project into the interface, destabilizing the dimer (Sukumaran et al., 2011). To gain further insight into the relationship between the interface stability and cleft dynamics, we analyzed two mutants generated in silico: L144D (GluA2), to destabilize the GluA2 interface via like-charge repulsion, and R163I (GluA3), to strengthen the labile GluA3 interface via hydrophobic contacts.

**Figure 8:**
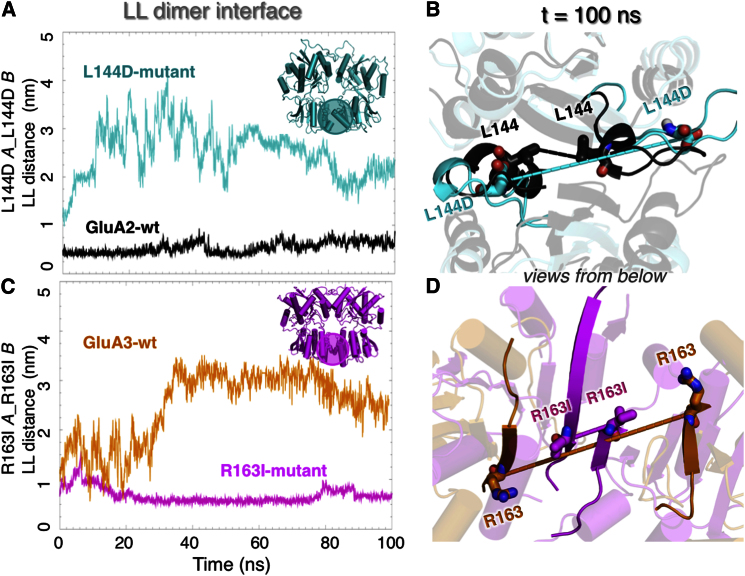
Critical Role of Interresidue Interactions at LL-LL Interface in Defining NTD Dimer Dynamics Results are presented for the mutants L144D (GluA2) and R163I (GluA3) to examine the significance of hydrophobic versus charged interactions in defining the distinctive dynamics of GluA3 and GluA2. (A) Time evolution of the closest interatomic distance between L144 residues on neighboring subunits for the wild-type (black) and between D144 pairs in the mutant (teal). Inset highlights the region of mutation. (B) Snapshots of wild-type GluA2 and L144D mutant at 100 ns, superimposed and viewed from bottom. (C and D) Same as (A) and (B) for GluA3 wild-type and mutant R163I. See also Figure S6 and Table S4.

Introduction of like-charges into the GluA2 LL interface indeed led to destabilization as can be seen from the comparison of the LL-LL distances for the mutant L144D (teal curve in Figure 8A) and for the wild-type GluA2 (black curve). Notably, the extent of destabilization is comparable to that originally observed for GluA3: interlobe distance between the two substituted amino acids increases to more than 30 Å within tens of nanoseconds, whereas in wt GluA2, the equivalent interaction is maintained over a period of 100 ns. Conversely, introducing hydrophobic residues into the GluA3 LLs leads to a more stabilized interface (Figure 8C): the distance between the mutated R163I residues is maintained but is disrupted in wt GluA3 at early stages of the simulation. Snapshot at *t* = 100 ns illustrates the disruption of the LL packing interface upon L144D mutation in GluA2 (Figure 8B) and strengthening in R163I (Figure 8D). These results thus demonstrate the stabilizing role of hydrophobic residues at the packing interface in GluA2, as well as the destabilizing role of buried arginines in GluA3. CM distances between the LLs (Figure S6A) further establish that the GluA2-L144D mutant weakens dimer contacts, whereas GluA3-R163I is stabilizing.

Analyses of the trajectories generated for the mutants show that LL stability is coupled to UL-LL dynamics: a salt bridge connecting the lobes of the clamshell (D98–K112; Figure S6B, expected to restrain cleft motions, is destabilized in the GluA2 mutant, whereas the R163I mutation stabilizes the equivalent salt bridge in GluA3 (D104–R141; Figure S6C). Moreover, stabilization of the LL in GluA3-R163I restricts clamshell motions as compared to the wt GluA3 dimer. Therefore, alterations of LL interface strength can propagate to the hinge region in both cases and has the capacity to alter lobe motions, that is, the perturbation of LL stability in both GluA2 and GluA3 has bidirectional effects that extend beyond local (LL) interactions.

## Discussion

In this study, we provide a series of insights into the dynamics of AMPA- and NMDA-receptor NTDs. First, we present the crystal structure of the GluA4 NTD, facilitating a comprehensive analysis of this sequence-diverse domain across the AMPAR subfamily. Second, we provide mechanistic insights into the intrinsic dynamics of GluN2B that facilitate ligand binding and offer a first glance into the motions driving GluN2B NTD allostery, whose *modus operandi* has not been elucidated to date. Third, we reveal that AMPAR- and NMDAR-NTD monomers share surprisingly similar global mode motions. These are restricted, but not abolished, upon dimerization in a subunit-dependent fashion, dictated by the evolutionary and structurally variable LL interface. Fourth, we show that AMPAR NTDs can readily reconfigure into NMDAR NTD conformers, a further indication of their unexpected similarity and their putative allosteric capacity. Fifth, we evaluate the dynamics of the AMPAR subfamily at atomic resolution, where, in accordance with experimental data, we find that GluA3 features the weakest LL dimer interface, which ruptures after ∼5 ns of MD simulations, followed by GluA3 LL secondary structure elements (αF) flipping downward to the LBD. The unique LL packing of GluA3 also potentiates it to undergo classic PBP-like clamshell motions. Finally, we capture critical residues at the LL-LL interface that mediate interprotomer dynamics in AMPARs, consolidated by analyses of mutants designed to weaken or strengthen the LL interface.

The AMPAR NTDs studied here are stable homodimers in solution, with a highly conserved UL interface, which will maintain dimer stability. The LL, on the other hand, which potentially shares an interface with the LBD, may play a mediatory role in the allosteric regulation, also demonstrated by the recent NMDAR NTD heterodimeric structure (Karakas et al., 2011). In AMPARs, helices αE and αF along with β7 together form the LL dimer interface in most crystal structures. Previous work has established that the GluA3 NTD assembles into homodimers less tightly packed and preferentialy coassembles with other AMPAR NTDs into heterodimers; the weak homomeric LL interface underlies the distinctive dynamics of the GluA3 NTD (Rossmann et al., 2011; Sukumaran et al., 2011). Indeed, in our simulations of all AMPAR NTD homodimers, GluA3 is distinguished by its high mobility: αE (L137–K151) shows considerable unwinding; αF tilts toward the NTD/LBD interface. A partial loss of helicity in αE is also observed in the recent structure of a kainate receptor (GluK3) NTD (Kumar and Mayer, 2010; Sukumaran et al., 2012), which also assembles as preferential heteromers, supporting the link between enhanced mobility (or lower stability) at the LL-LL interface and low homodimeric assembly propensity. The instability of the GluA3 LL dimer interface may propel toward the UL interface, which is apparent in MD simulations (Figure S5). The observed loosening of the hydrophobic core (F56 and F88) may facilitate interprotomer rotations. The downward motion of helix αF toward the LBD in MD trajectories suggests a potential role in the allosteric propagation of NTD motions. The crosstalk between NTD and LBD will also be affected by the connecting linker. This segment is sequence variable between the paralogs and harbors two N-glycosylation sites. These have been removed in the GluA2 homomeric structure (PDB ID code 3KG2) along with a deletion encompassing six residues (Sobolevsky et al., 2009). How this mutation affects domain packing and allosteric communication in iGluRs is a key open question.

Our analysis reveals the ability of individual protomers to undergo concerted clamshell opening/closing motions, which simultaneously affect interprotomer contacts. This supports a possible cooperative response of AMPAR NTDs upon ligand binding or interaction with protein partners (O'Brien et al., 1999) and a capacity to transmit signals toward the channel. We note that ligand interaction may not be restricted to the interlobe cleft but could target the LL dimer interface, as known from analogous cases (He et al., 2006; Mony et al., 2011), or the highly dynamic αH region.

The NTD of the NMDAR is known to modulate channel gating by binding Zn^2+^ ions and ifenprodil-like compounds, thereby sparking clinical interest in these domains. The twisted LL along with surface properties (Karakas et al., 2009; Stroebel et al., 2011) have been purported to be the primary reason why NTD-mediated modulation of the ion channel is seen in GluN2B and NMDAR heterodimers but have not been seen so far in non-NMDA receptors. Structural dynamics analysis offers a different perspective, where the global motions accessible to the different NTD structures of the iGluR families overlap remarkably. Also, the global modes of dimeric AMPAR, NMDAR, and mGluR NTDs allow for facile transitions from one form to another, suggesting that the AMPAR NTDs may equally have allosteric signaling abilities.

Binding of ions and small molecules to the NTD are most likely facilitated by global motions in NMDARs. NTD clamshell motions have been implicated in facilitating an induced-fit binding mechanism (Karakas et al., 2011). Based on similarity of global motions between AMPARs and NMDARs, the allosteric effect known to modulate NMDAR open probability should not be disregarded for non-NMDARs. This view is further strengthened by the small-molecule binding capacity in the GluA2 cleft reported previously (Sukumaran et al., 2011; Cameron et al., 2012) and the labile nature of the GluA3 NTDs observed here. The present analysis is a further step toward clarifying the putative allosteric potential of AMPAR NTDs by highlighting their intrinsic ability to undergo motions comparable to NMDAR NTDs and their propensity to sample conformers observed in NMDARs. This opens an avenue of searching for molecules able to bind AMPAR NTDs, which may in turn play an important role in regulating gating of nonNMDA iGluRs.

## Experimental Procedures

### Protein Crystallography

GluA4 NTD constructs (Ala1–Asp380; all residue numbers correspond to the sequence of the mature protein, after signal peptide cleavage) were designed, expressed, and purified as described previously (Rossmann et al., 2011). Crystallization was performed using the vapor-diffusion method (Benvenuti and Mangani, 2007). GluA4 NTD readily crysallized in 0.1 M sodium cacodylate and 0.7–1.0 M citrate (pH 5.0–7.0). Diffraction data were collected from beamline I03 at the Diamond Light Source (Oxford, UK). Data were processed using the iMOSFLM package (Battye et al., 2011). The structure was solved by molecular replacement with PHASER (McCoy et al., 2007), using a GluA2 NTD monomer (PDB ID code 3HSY, chain *B*) as a search probe. The model was alternately refined using REFMAC (Murshudov et al., 2011) and manually rebuilt in COOT (Emsley et al., 2010). MOLPROBITY (Davis et al., 2007) was used to validate model stereochemistry.

### MD Simulations

The GROMACS program (Van Der Spoel et al., 2005) was used to generate MD trajectories for the systems listed in Table S4. The proteins were solvated with single point charge (Berendsen et al., 1981) water molecules, using GROMOS 43a1 force field (Lindahl et al., 2001). MD runs were performed at 310 K (by implementing Berendsen's temperature coupling to protein and water molecules) and atmospheric pressure. Electroneutrality was achieved by adding counterions. Electrostatic interactions were treated with the particle mesh Ewald method (Darden and Pedersen, 1993) and the LINCS (Hess et al., 1997) algorithm was used to constrain the bond lengths, enabling an integration timestep of 2 fs. Each system was energy minimized using the steepest descent algorithm, followed by an equilibration of 2 ns, before the productive runs of 100 ns. During equilibration, backbone atoms were restrained by harmonic potentials, while side-chain atoms and water molecules were allowed to relax. Figure S7 displays the rmsds from the initial state averaged over all residues as a function of time for the four AMPAR NTD subtypes.

### ANM Analysis of Collective Motions

NMA with ANM was performed as previously described (Atilgan et al., 2001; Eyal et al., 2006). In the ANM, the overall potential (V_ANM_) is represented as the sum of harmonic potentials between interacting nodes. Those nodes within a certain cutoff distance (15 Å) are assumed to interact. The force constants (the second derivatives of the potential V_ANM_) for the 3*N*×3*N* interactions (for *N* residues in 3D) are given by the elements of the Hessian matrix H (a summation over interresidue pairwise potentials with uniform force constants). The method yields a unique set of collective modes for each protein, represented by 3*N*-dimensional eigenvectors *u*^*(k)*^ (1 ≤ *k* ≤ 3*N*-6) obtained by decomposing the Hessian matrix H. The eigenvalues λ_*k*_ of H scale with the square frequencies and λ_*k*_^−1/2^ defines the weight of mode *k* such that the conformational change due to mode *k* scales with λ_*k*_^−1/2^u ^*(k)*^; the square displacements (Figures 2, 4, and 5) scale with the λ_***k***_^−1^.

### Overlap between Experimentally Observed Structural Changes and Soft Modes

Consider two known structures S1 and S2 for the protein (family) of interest, represented each by conformational vectors {R_*0*_}_A_ and {R_*0*_}_B_. Their structural difference (after optimal alignment using STRAP; Gille and Frömmel, 2001) is Δ{R}_S1_→_S2_ = {R_*0*_}_S2_ − {R_*0*_}_S1_. To observe if S1 is predisposed to undergo this change, we evaluate the *overlap* or correlation cosine, (*d*. *u*^*(k)*^) for a subset of soft modes *u*^*(k)*^ accessible to S1. Here, *d* is the unit vector along Δ{R}_S1_→_S2_. The cumulative overlap achieved by a subset of *m* modes is given by CO(*m*) = [Σ_*k*_ (*d*. *u*^*(k)*^)^2^]^1/2^, where the summation is performed over 1 ≤ *k* ≤ *m*. The complete set of eigenvectors forms an orthonormal basis, that is, CO(*m*) = 1 for *m* = 3*N*-6.

### NMA of a Subsystem Coupled to an Environment

The dynamics of a system (S) in the context of an environment (E) is evaluated by partitioning H into four submatrices (Zheng and Brooks, 2005; Ming and Wall, 2005a)

(1) $$H=\left(\begin{array}{cc} H_{\text{SS}} & H_{\text{SE}}\\ H_{\text{ES}} & H_{\text{EE}} \end{array}\right),$$

where H_SS_ refers to interactions within the system, H_EE_ to those within the environment, and H_SE_ (or H_ES_) to the coupling between S and E. The resulting effective Hessian of the system is in the presence of the environment

(2) $$H_{\text{SS}}^{\text{eff}}=H_{\text{SS}}-H_{\text{SE}}H_{\text{EE}}^{-1}H_{\text{ES}}.$$

(A) and (B) in Figures 5 and S2 are obtained by evaluating the correlation cosine [*u*^*(k)*^. *u*_*eff*_^*(l)*^] between the eigenvectors *u*^*(k)*^ to *u*_*eff*_^*(l)*^ (for *k, l* = 1–40) corresponding to H_SS_ and H^eff^_SS_, respectively.

## Figures and Tables

**Table 1:** Crystallographic Data Collection and Refinement Statistics

	GluA4-NTD
X-ray source	IO3, Diamond
Wavelength (Å)	0.9393
Space group	P4_3_2_1_2
Unit cell parameters	*a* = 104.54, *b* = 104.54, *c* = 108.98; α = β = γ = 90°
Resolution range (Å)	54.49–2.25 (2.308–2.25)^a^
Observed reflections	205,233 (30,373)
Unique reflections	29,299 (4,210)
Completeness (%)	99.9 (100.0)
Multiplicity	7.0 (7.2)
Mean I/σ(I)	12.5 (3.0)
R_merge_	0.102 (0.660)
Average mosaicity	0.37

Reflections in test set	1,484
R_work_	0.1866
R_free_	0.2405
Protomers per ASU	1
Number of residues	380
Number of waters	209
Number of non-protein molecules^b^	5
Wilson B-factor (Å^2^)	45.35
Mean protein B-factor (Å^2^)	45.29
Mean water B-factor (Å^2^)	51.06
Mean non-protein^b^ B-factor (Å^2^)	71.03
RMSD from ideal bond length (Å)	0.022
RMSD from ideal bond angle (°)	1.966

See also Figure 1.

a Values in parentheses are for the highest resolution shell.

b Carbohydrate residues from *N*-glycosylation.

## Accession Numbers

The Protein Data Bank accession number for the GluA4 structure reported in this paper is 4GPA.

## Supplemental Information

Document S1. Figures S1–S7 and Tables S1–S4

Movie S1. Global Mode of Motion of all AMPAR dimeric NTDs, Related to Figure 2

Movie S2. Twisting Motion in Mode 1 of AMPAR Monomers, Related to Figure 4

Movie S3. Twisting Motion in Mode 1 of NMDAR Monomers, Related to Figure 4

Movie S4. Clamshell Motion in Mode 2 of AMPAR Monomers, Related to Figure 4

Movie S5. Clamshell Motion in Mode 2 of NMDAR Monomers, Related to Figure 4

Movie S6. Transition from Dimeric Conformer of NMDA-GluN1 to AMPA GluA2 NTD Dimer Conformer, Related to Figure 6

Movie S7. Transition from Dimeric Conformer of AMPA GluA3 to Heterodimeric NMDAR NTD, Related to Figure 6

Movie S8. GluA3 100 Nanosecond MD Simulation, Related to Figure 7
