# Supplementary material for: Comparative Dynamics of NMDA- and AMPA-Glutamate Receptor N-Terminal Domains
Source: Structure. 2012 Nov 7;20(11):1838–49. doi: 10.1016/j.str.2012.08.012 (PMC3496038; doi:10.1016/j.str.2012.08.012)
Supplement: Document S1. Figures S1–S7 and Tables S1–S4 [file mmc1.pdf]

Structure, Volume *20*

Supplemental Information

Comparative Dynamics of NMDA- and AMPA-Glutamate Receptor N-Terminal Domains

Anindita Dutta, Indira H. Shrivastava, Madhav Sukumaran, Ingo H. Greger, and Ivet Bahar

Inventory of Supplemental Information

Figure S1– Relates to Figure 1

Figure S2 – Relates to Figure 5

Figure S3 – Relates to Figure 6

Figures S4 and S5 – Relate to Figure 7

Figure S6 – Relates to Figure 8

Figure S7 – Relates to Figure 7 and MD simulation methods

Table S1 – Relates to Figures 1 and 2

Table S2 – Relates to Figures 2 and 4

Table S3 – Related to Figures 2 and 4

Table S4 – Related to Figures 3, 7 and 8

Movie S1 –Relates to Figure 2

Movie S2 and S3 –Relate to Figure 4

Movies S4 and S5 – Relate to Figure 4

Movie S6 – Relates to Figure 6

Movie S7 – Relates to Figure 6

Movie S8 – Relates to Figure 7

Supplementary Figures

Supplementary Figure S1. (*Relates to Figure 1*)

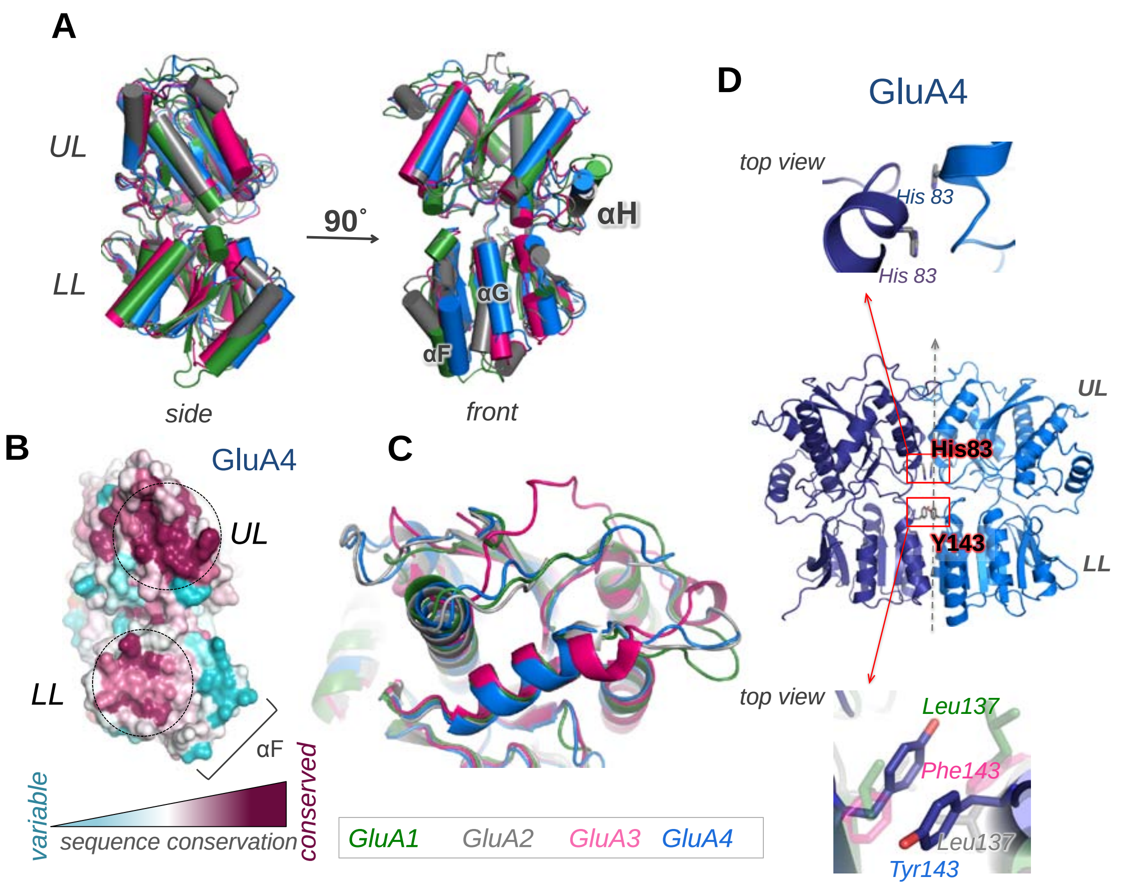

Supplementary Figure S1. Comparative structural analysis of AMPAR NTDs. (A) Structural superposition of all four AMPAR NTD monomers shown in side and front views (GluA1: PDB 3SAJ, *green*; GluA2: PDB 3HSY, *grey*; GluA3: PDB 3O21, *red*; GluA4 (new structure), *blue*). (**B**) Dimeric interface of GluA4, colored by position-specific patterns of conservation (generated by the ConSurf package (Ashkenazy H et al, 2010) and a manually curated alignment of 142 AMPAR paralogs. The UL interface is more conserved than the LL interface **(C)** Focuses on the superposition of the top loops that show some degree of variability in all AMPAR NTD paralogs. **(D)** Shows the difference in interfacial packing of two critical residues in GluA4 compared to other AMPAR counterparts.

Supplementary Figure S2. (*Relates to Figure 5*)

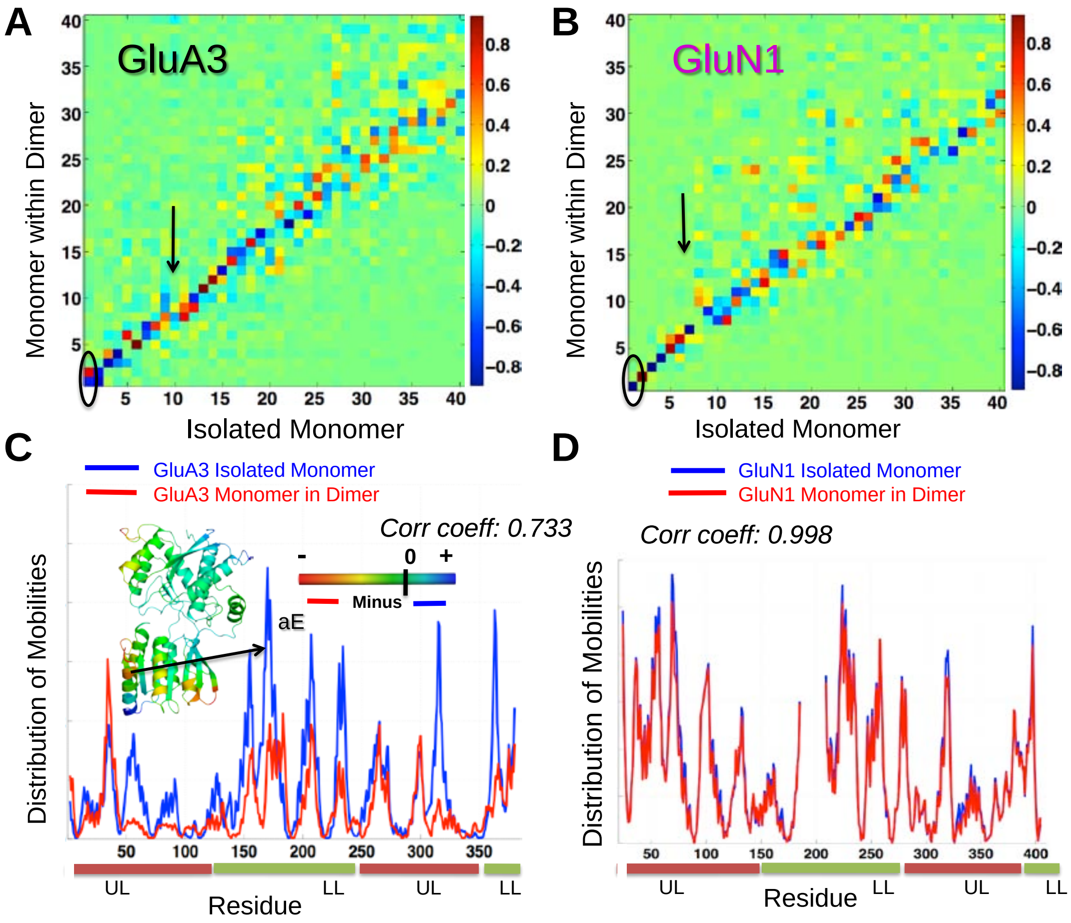

Supplementary Figure S2. Effect of heterodimerization on NTD monomer dynamics of AMPA and NMDA receptors (A) Correlation cosine between the top 40 eigenmodes accessible to the isolated GluA3 protomer (3O21-*C*; *abscissa*) and the same protomer in the dimer (3O21-*CD*; *ordinate*). The encircled region shows that isolated monomer *mode 1* is accounted by the *2nd mode* in the dimer. (**B**) Same as (**A**), for NMDA GluN1 (3QEL-*C*) isolated monomer compared to its behavior in the heterodimer (3QEL) (**C, D**) Mobility profiles for GluA3 and GluN1 monomers in isolation and in their respective dimers, showing the suppression of mobilities upon dimerization in both AMPAR GluA3, but not in GluN1 that retains its flexibility on dimerization. The inset in **(C)** shows GluA3 monomer colored by difference in mobility i.e., red curve – blue curve. The color bar indicates that red implies + values or regions where monomer motion is most suppressed in the monomer on dimerization and blue showing regions where motion is suppressed in the isolated form.

Supplementary Figure S3. (*Relates to Figure 6*)

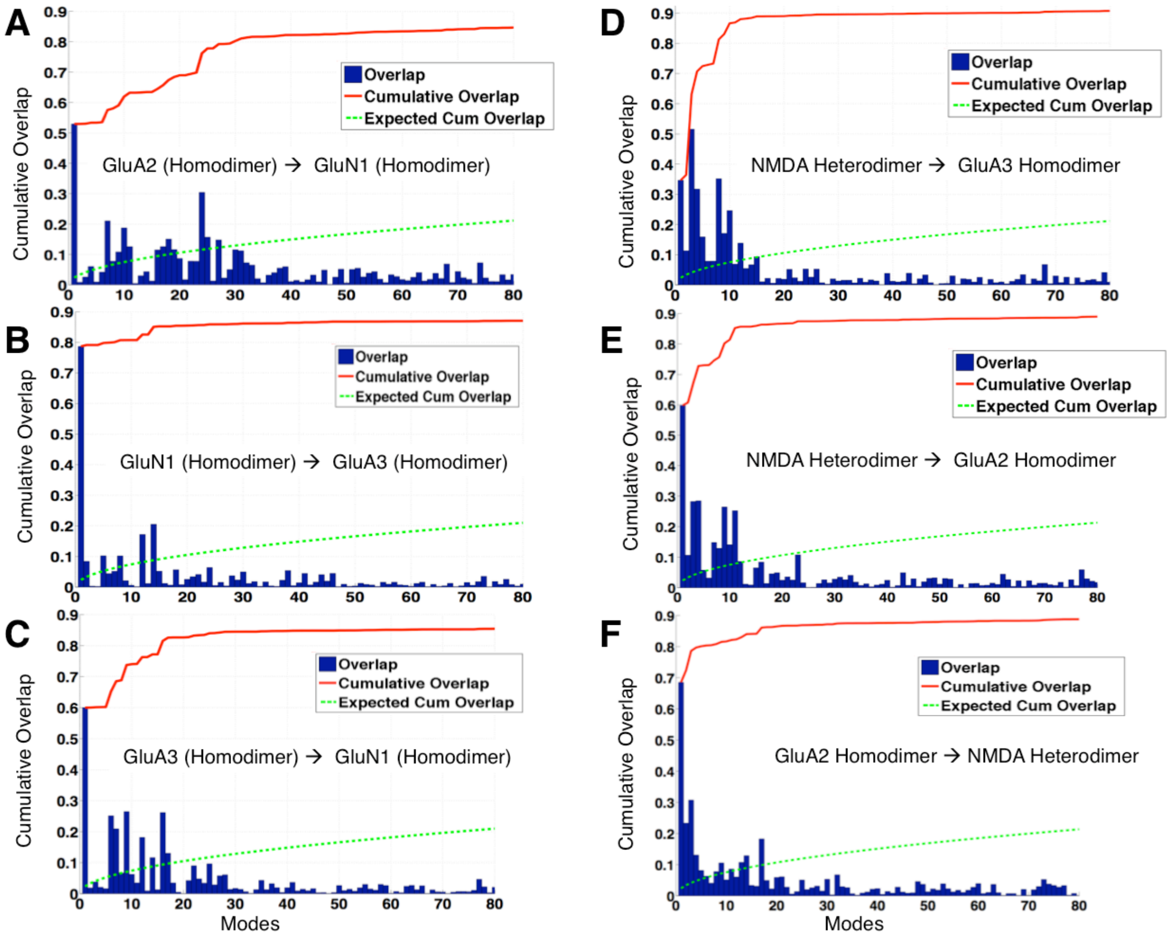

Supplementary Figure S3. Ease of transitions between dimeric conformers of NMDA and AMPA receptors NTDs. (A) The back transition from GluA2 to GluN1 is shown here and can also be explained by ANM modes (90% using 80 modes). **(B** and **C)** show the transition from dimeric GluN1 to GluA3 and back from GluA3. **(D)** Shows transition from NMDA heterodimer to GluA3, the top 80 modes of the NMDA receptor can achieve 90% of the deformation **(E)** Shows the transition from NMDA heterodimer to GluA2 and **(F)** from GluA2 to NMDA heterodimer.

Supplementary Figure S4. (*Relates to Figure 7*)

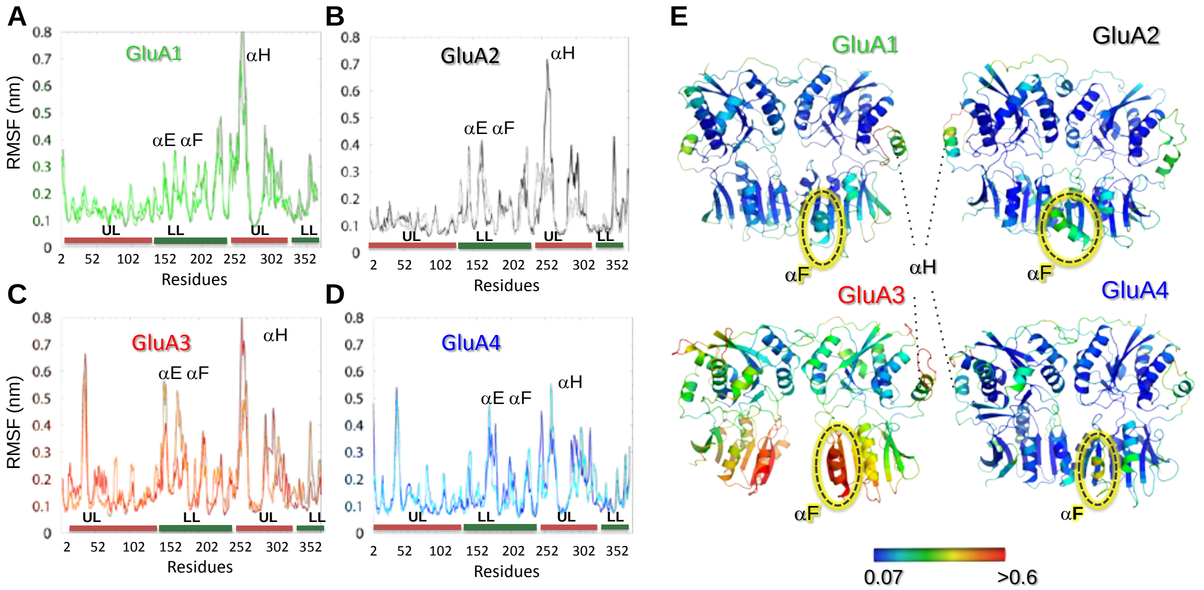

Supplementary Figure S4. Fluctuation profiles of residues for AMPAR NTDs. (A-D) RMSFs plotted as a function of residue index. The helices with high mobilities (αE, αF and αH) are labelled. We also note the suppressed motions of GluA1 and GluA2 at the UL N-terminal portions. GluA3 and GluA4 exhibit peaks near A36-T37 (on the loop between αA and β2; not labeled). The bars below the curves in **A-D** indicate the UL (*red*) and LL (*green*) segments along the sequence. In panel **E**, the respective structures are color-coded according to their residue RMSF values plotted in panels **A-D**, from least mobile (*blue*) to most mobile (*red*). The most mobile helix, αH, is indicated. The helix αF is encircled to highlight its high mobility.

Supplementary Figure S5. (*Relates to Figure 7*)

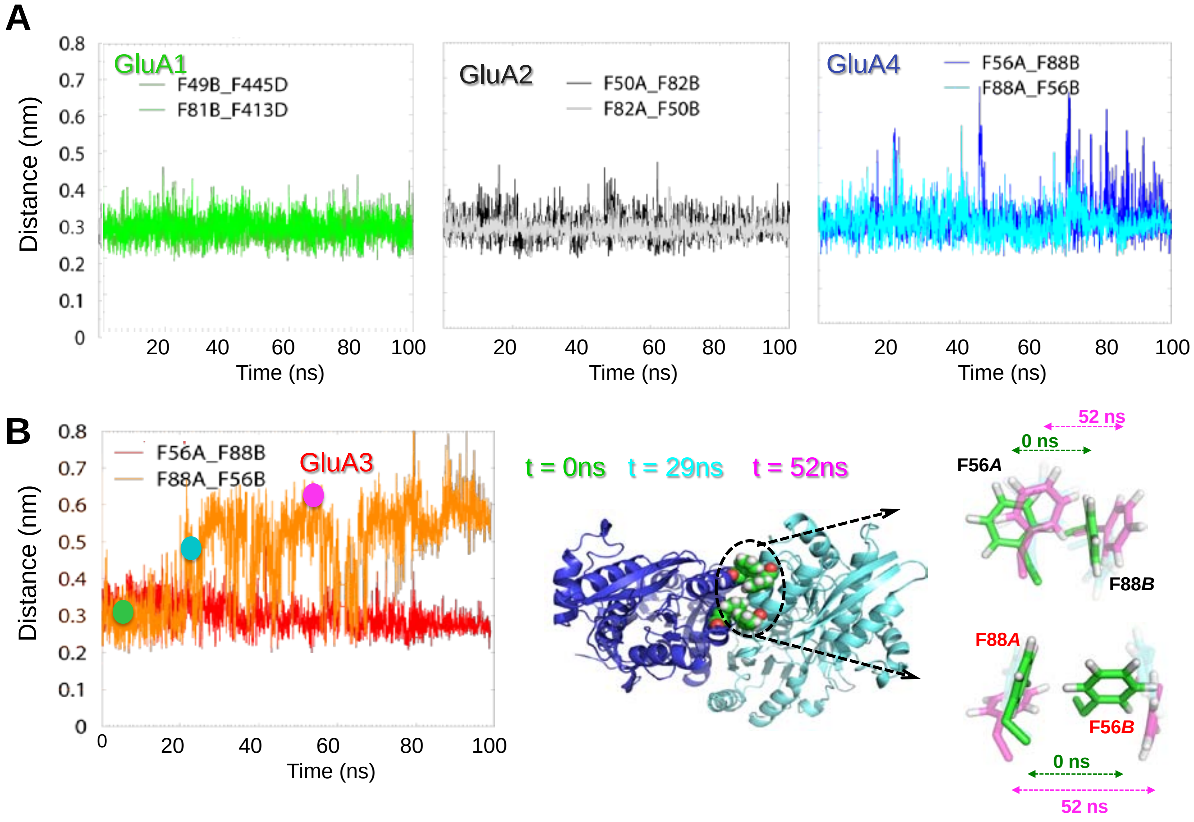

Supplementary Figure S5. UL interface stabilities for GluA1-4 (A) We show the distances measured between Phe residues at the UL interfaces, between the two protomers, in each of GluA1, GluA2 and GluA4. There are two pairs of the Phe-Phe interaction at the UL interface, which are both maintained in GluA1, GluA2 and GluA4. (**B**) The same distance is shown for GluA3. In GluA3, one of the interaction weakens at ~30ns, after which it exhibits large fluctuations. The snapshots of the interacting pairs of Phe (F56*A*_F88*B*, and F88*A*_F56*B*) in GluA3 are illustrated at t=0ns, 29ns and 52ns. While the F56*A*_F88*B* interaction is maintained, the F88*A*_F56*B* interaction is disrupted, mainly due to movement of F56*B*.

Supplementary Figure S6. (*Relates to Figure 8*)

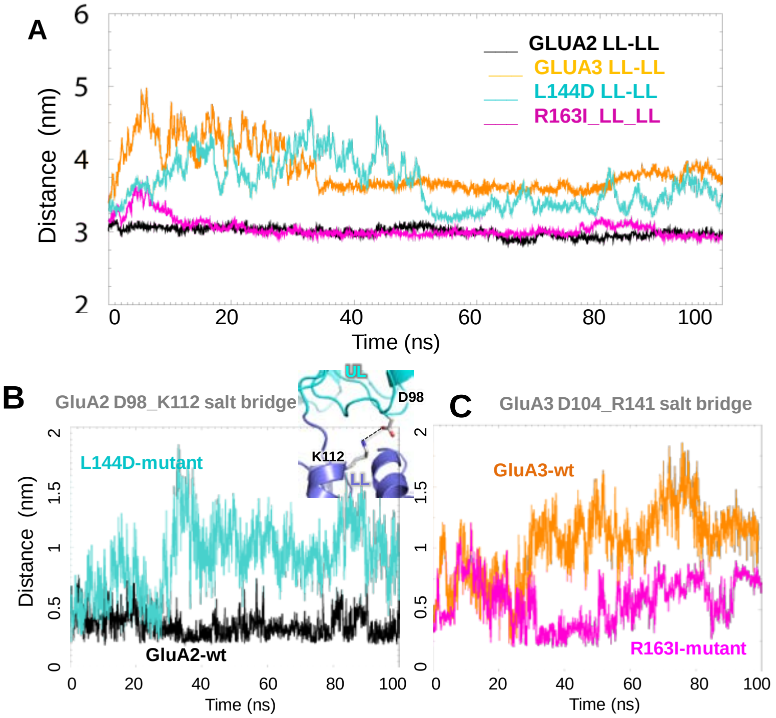

Supplementary Figure S6. Time evolution of interlobe distance observed for wild type and mutant GluA2 and GluA3. (**A**) Distance between the LL mass centers for GluA2 (*black*), GluA3 (*orange*), GluA2 mutant (L144D) (*teal*) and GluA3 mutant (R163I) (*magenta*). GluA2 WT and GluA3 mutant R163I are highly stable, indicated by the constant distance maintained, at ~ 3nm. On the other hand, the destabilizations of the LL interface in GluA3 WT and GluA2 mutant L144D are evident during the early stages of the simulation, lasting up to ~ 40ns. (**B**) Distance between UL-LL salt-bridge forming residue bresidues pairs D98 and K112 for GluA2 (*black*) and GluA2 L144D mutant (*teal*). The distance is measured in terms of the minimum distance between any two atoms of the two residues. (**C**) same as (**B**) between UL D104 and LL R141 for GluA3 (*orange*) and GluA3 R163I mutant (*magenta*).

Supplementary Figure S7. (*Relates to Figure 7*)

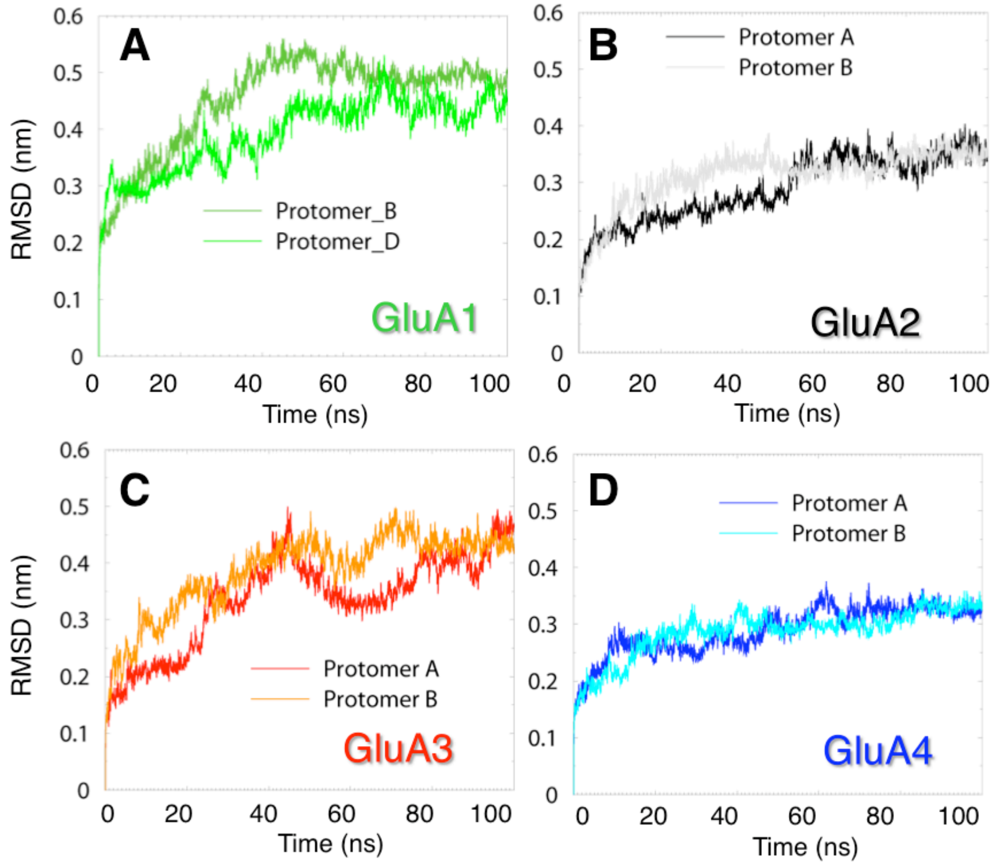

Supplementary Figure S7. Comparison of the time evolution of RMSDs in residue positions for AMPAR NTDs. Results are shown for both protomers in (**A**) GluA1 (**B**) GluA2 and (**C**) GluA3, and (**D**) GluA4. A departure of ~0.35 nm from the starting structure is observed in GluA2 and GluA4 protomers. In GluA3 this value reaches ~0.47nm (originating from the enhanced mobility of the LL helices αE and αF, in particular) and in GluA1, 0.55nm (due to large mobility of the αH region).

Supplementary Tables

Supplementary Table S1. *(Relates to Figure 1,2)*
RMSD values (Å) between different AMPAR NTD dimeric structures

RMSD	GluA1-*AC*	GluA1-*BD*	GluA2-*AB*	GluA3-*AB*	GluA3-*CD*	GluA4-*BA*
GluA1-*AC*	-	0.815	1.019	3.382	2.901	1.264
GluA1-*BD*		-	1.067	3.323	2.864	1.282
GluA2-*AB*			-	3.418	3.333	0.991
GluA3-*AB*				-	4.094	3.163
GluA3-*CD*					-	2.430
GluA4-*BA*						-

**AMPAR NTD structures exhibit different types of protomer-protomer packing. The identifiers in italic refer to the labels of the chains in the respective PDB files 3SAJ, 3HSY, 3O21 for GluA1-GluA3. The PDB file for GluA4 (currently resolved) is not yet available.*

Supplementary Table S2. (*Relates to Figure 2,4*)
Correlations between the global motions favored by iGluR subfamily NTDs*

**A. Correlations between the first global mode of AMPAR NTD dimer subtypes**						
		**AMPAR NTD Dimers**				
		GluA1-*AC*	GluA1-*BD*	GluA2-*AB*	GluA3-*CD*	GluA4-*BA*
**AMPAR NTD Dimers**	GluA1-*AC*	-	0.983	0.897	0.876	0.922
	GluA1-*BD*		-	0.916	0.912	0.939
	GluA2-*AB*			-	0.874	0.926
	GluA3-*CD*				-	0.926
	GluA4-*BA*					-

**B. Correlations between global modes of AMPAR and NMDAR NTD protomers**					
		**NMDAR NTD Monomers**			
		GluN1-*A* (Mode 1)	GluN1-*A* (Mode 2)	GluN2B-*A* (Mode 1)	GluN2B-*A* (Mode 2)
**AMPAR NTD Monomers**	GluA1-*A*	0.757	0.756	0.731	0.757
	GluA2-*B*	0.765	0.672	0.816	0.628
	GluA3-*C*	0.734	0.713	0.723	0.675
	GluA4-*A*	0.841	0.823	0.796	0.824

(*) Correlations are evaluated as the cosines between the 3N-dimensional eigenvectors corresponding to the softest modes of collective motions predicted by the ANM

Supplementary Table S3. (*Relates to Figure 2,4*)
Relative sizes of NMDAR and AMPAR NTD global motions (*)

**AMPAR NTD Dimers**						
GluN1<br>*AB*	GluN2B-N1<br>*CD*	GluA1<br>*AC*	GluA1<br>*BD*	GluA2<br>*AB*	GluA3<br>*CD*	GluA4<br>*BA*
Mode 1						
1	0.87	0.25	0.27	0.24	0.42	0.25

**AMPAR -NMDAR NTD Monomers**					
GluN1-*A*	GluN2B-*A*	GluA1-*A*	GluA2-*B*	GluA3-*C*	GluA4-*B*
Mode 1					
1	0.58	0.54	0.52	0.59	0.52
Mode 2					
1	0.48	0.53	0.45	0.48	0.43

(*) based on the eigenvalues of the softest modes predicted by the ANM, normalized with respect to GluN1-A in case of monomers and GluN1-AB in case of dimers

Supplementary Table S4. (*Relates to Figures 3,7,8*)
Details of MD simulation systems

Structure	PDB-ID	Resolution	No. of Atoms	Simulation Time[a]
NMDA1	3JPW	2.80Å	78,095	50ns (0.47)
NMDA2	3JPY	3.21Å	78,066	50ns (0.35)
GluA1-*BD*	3SAJ	2.5Å	76,167	100ns (0.55)
GluA2	3HSY	1.75Å	75,262	100ns (0.35)
GluA3 -*CD*	3O21	2.2Å	76,345	100ns (0.47)
GluA4	Unpublished		76,231	100ns (0.35)
L144D	Model		76,257	100ns (0.45)
R163I	Model		76,324	100ns (0.45)

[a] The values in parenthesis correspond to average RMSDs from the starting structure.

Supplementary Movies

Supplementary Movie S1: (*Relates to Figure 2*)
Global Mode of Motion of all AMPAR dimeric NTDs. The torsional counter-rotation of the two protomers in *Mode 1* is displayed for GluA4 (currently resolved structure) as a representative structure for AMPAR NTD dimers. The ribbon diagram is colored by mobility (*red*: most mobile, *blue*: least mobile).

Supplementary Movies S2 and S3: (*Relates to Figure 4*)
Twisting motion in *Mode 1* of AMPAR and NMDAR monomers. Motion of GluA2 (3HSY-*B*) protomer (**Supplementary Movie S2**) and GluN2B (3JPY) in *Mode 1* (**Supplementary Movie S3**) as representative structures showing the counter-rotation motion of the two lobes that is seen in all AMPAR and NMDAR sub-types. The ribbon diagram is color-coded by mobility (*red*: most mobile, *blue*: least mobile).

Supplementary Movies S4 and S5: (*Relates to Figure 4*)
Clamshell motion in *Mode 2* of AMPAR and NMDAR monomers. The clamshell motion of GluA2 (3HSY-*B*) protomer (**Supplementary Movie S4**) and GluN2B (3JPY) in Mode 2 (**Supplementary Movie S5**) as representative structures showing the opening and closing of the two lobes (clamshell-like) that is seen in all AMPAR and NMDAR sub-types. The ribbon diagram is color-coded by mobility (*red*: most mobile, *blue*: least mobile).

Supplementary Movie S6: (*Relates to Figure 6*)
Transition from dimeric conformer of NMDA – GluN1 to AMPA GluA2 NTD dimer conformer. Motion of NMDA-GluN1 homodimer (3Q41) along *Mode 1* colored by mobility in that mode. The transparent structure in pink is GluA2 (3HSY) or the target structure. The mode shows how GluN1 approaches GluA2-like structure.

Supplementary Movie S7: (*Relates to Figure 6*)
Transition from dimeric conformer of AMPA GluA3 to heterodimeric NMDAR NTD. The motion of GluA3 (3O21-*CD*) along *Mode 1* colored by its mobility in that mode. The transparent structure in orange is NMDA heterodimer (3QEL) or the target structure. The ligand in magenta is ifenprodil bound at the interface of the NMDA heterodimer. The mode shows how GluA3 approaches the ligand-bound NMDA heterodimeric structure.

Supplementary Movie S8: (*Relates to Figure 7*)
GluA3 100 nanosecond MD simulation. Shows the trajectory in 100ns of MD simulations in GluA3. The helical regions of the protein are represented as cylinders, with the two protomers *C* and *D*, colored *red* and *orange*, respectively. The top loop Leucines (L310) and the UL interface residues F56 and F88 are represented in space-filling. The helix F in protomer D is highlighted in *purple* to better visualize its downward motion.

Reference:

1. Ashkenazy, H.,*et al.* ConSurf 2010: calculating evolutionarily conservation in sequence and structure of proteins and nucleic acids. *Nuc. Acids. Res*. **38**, W529-33.
