# Supplementary figures and images for: Comparative Dynamics of NMDA- and AMPA-Glutamate Receptor N-Terminal Domains
Source: Structure. 2012 Nov 7;20(11):1838–49. doi: 10.1016/j.str.2012.08.012 (PMC3496038; doi:10.1016/j.str.2012.08.012)

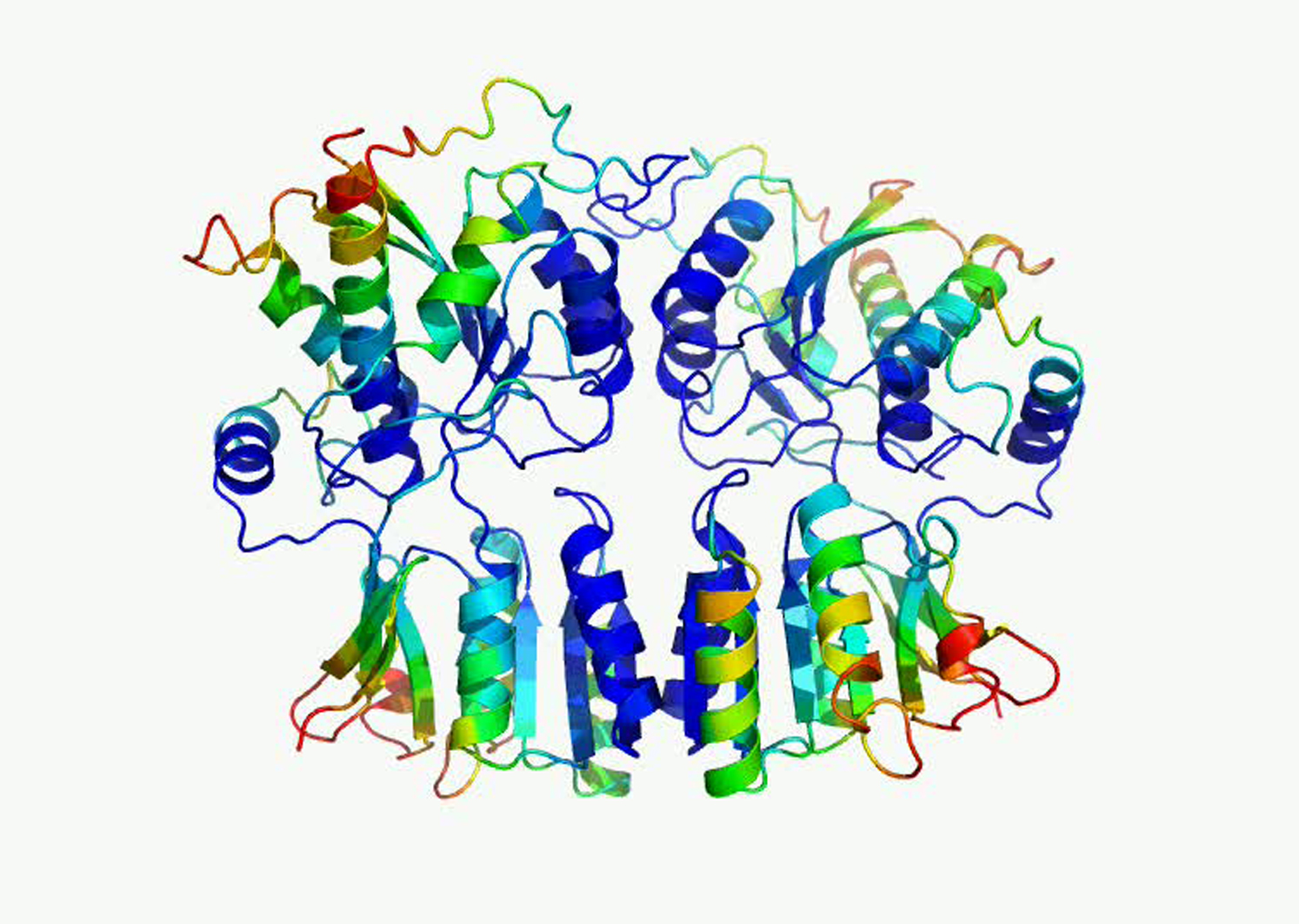

Supplement: Movie S1. Global Mode of Motion of all AMPAR dimeric NTDs, Related to Figure 2 [file mmc2.jpg]

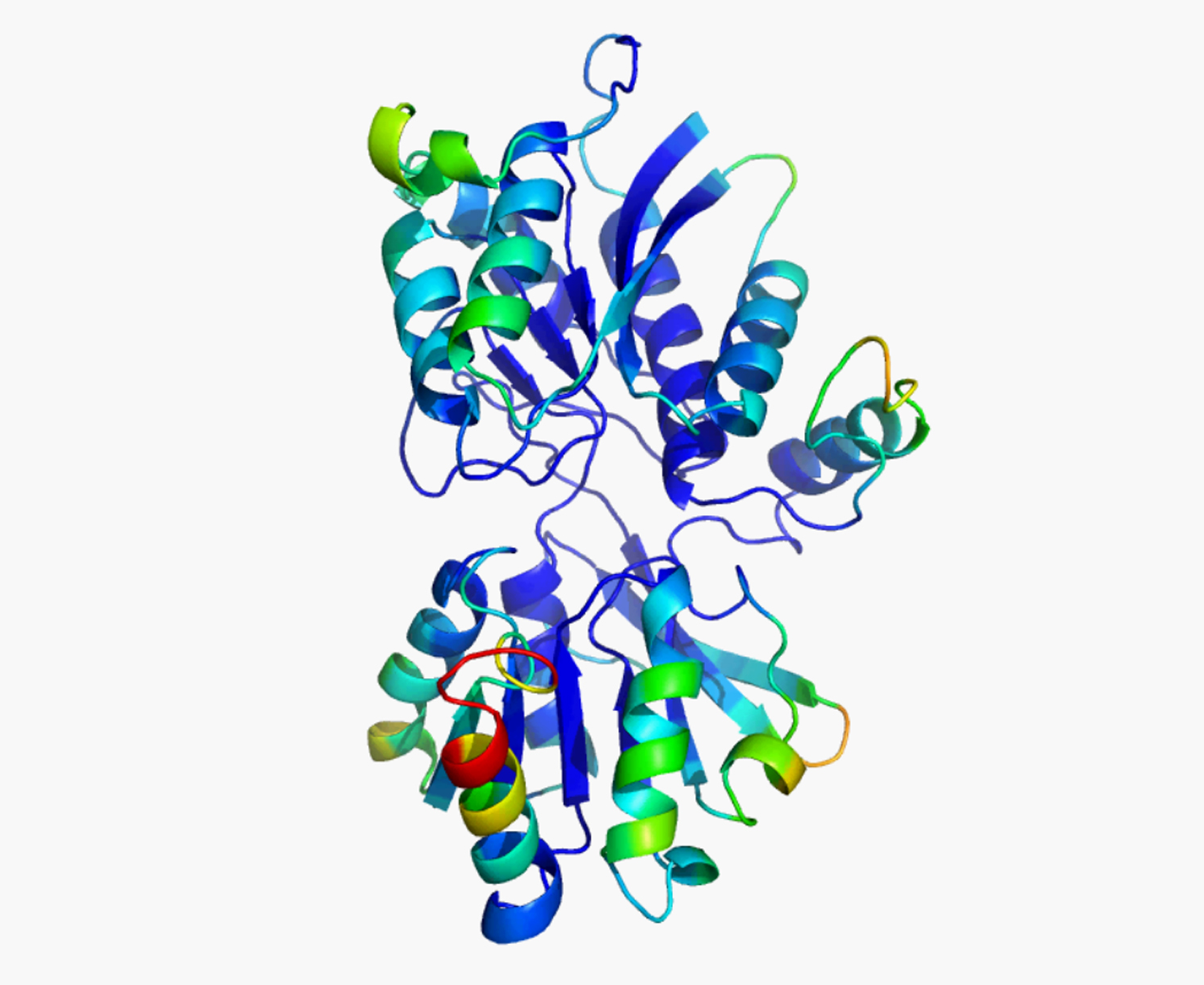

Supplement: Movie S2. Twisting Motion in Mode 1 of AMPAR Monomers, Related to Figure 4 [file mmc3.jpg]

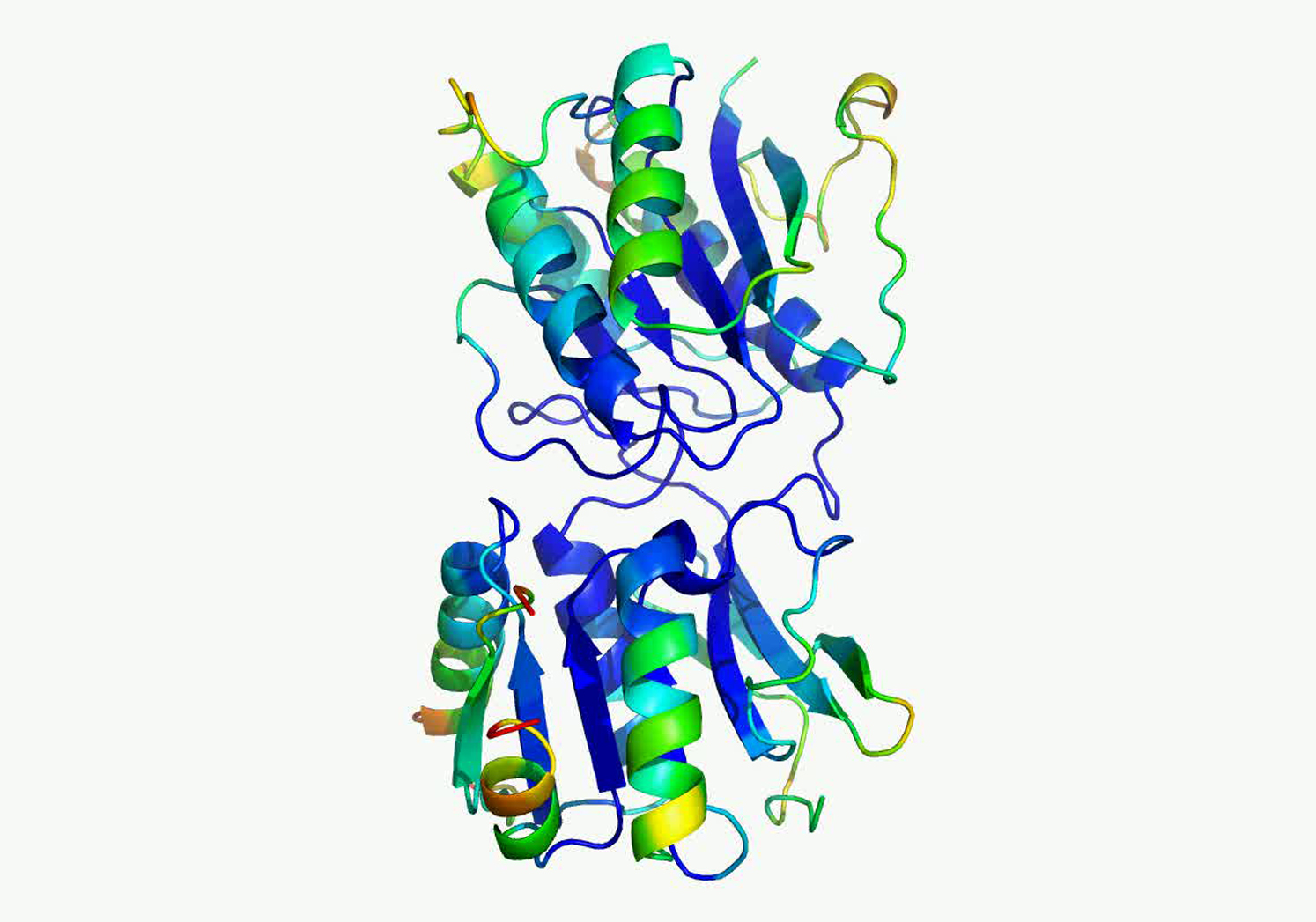

Supplement: Movie S3. Twisting Motion in Mode 1 of NMDAR Monomers, Related to Figure 4 [file mmc4.jpg]

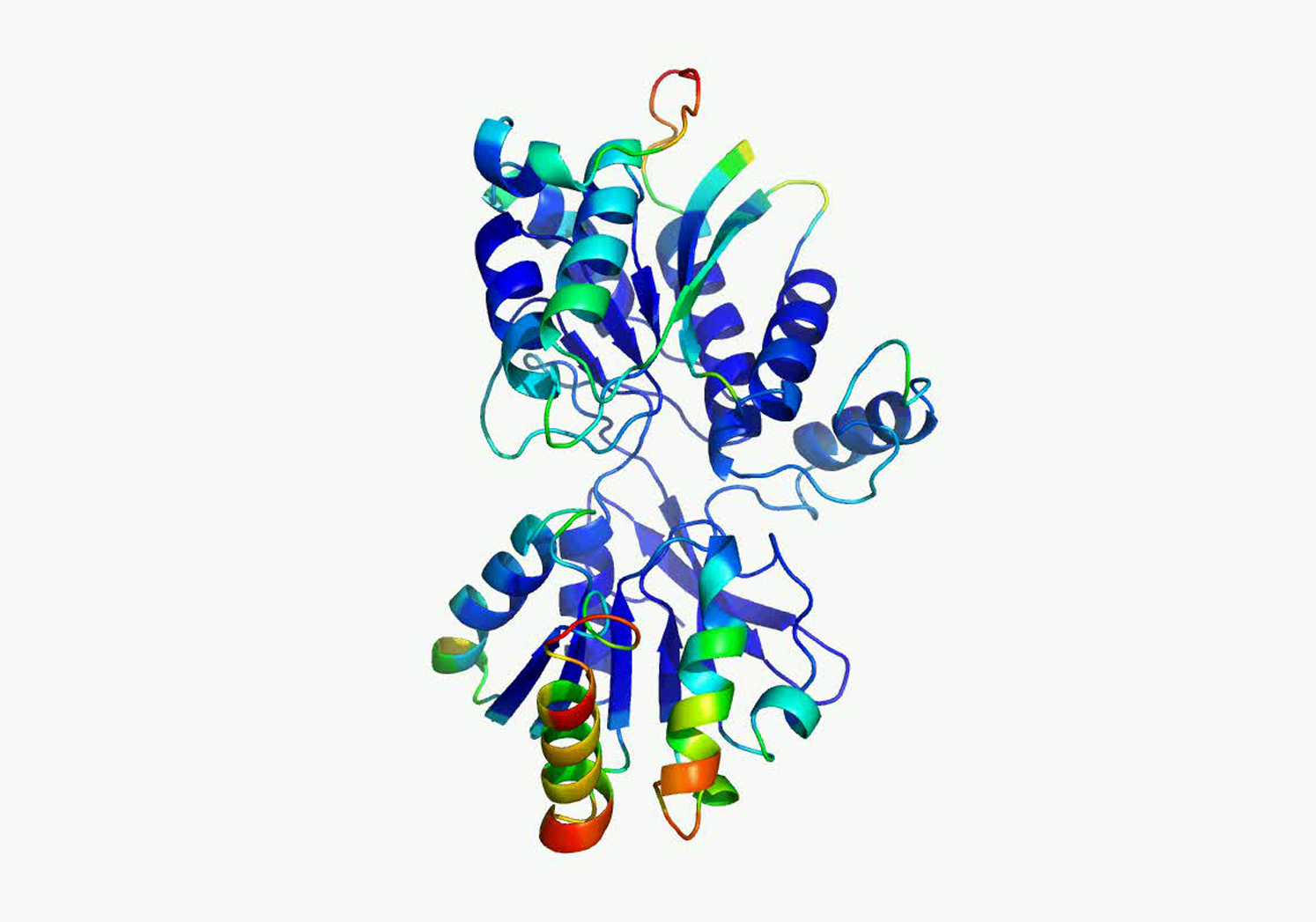

Supplement: Movie S4. Clamshell Motion in Mode 2 of AMPAR Monomers, Related to Figure 4 [file mmc5.jpg]

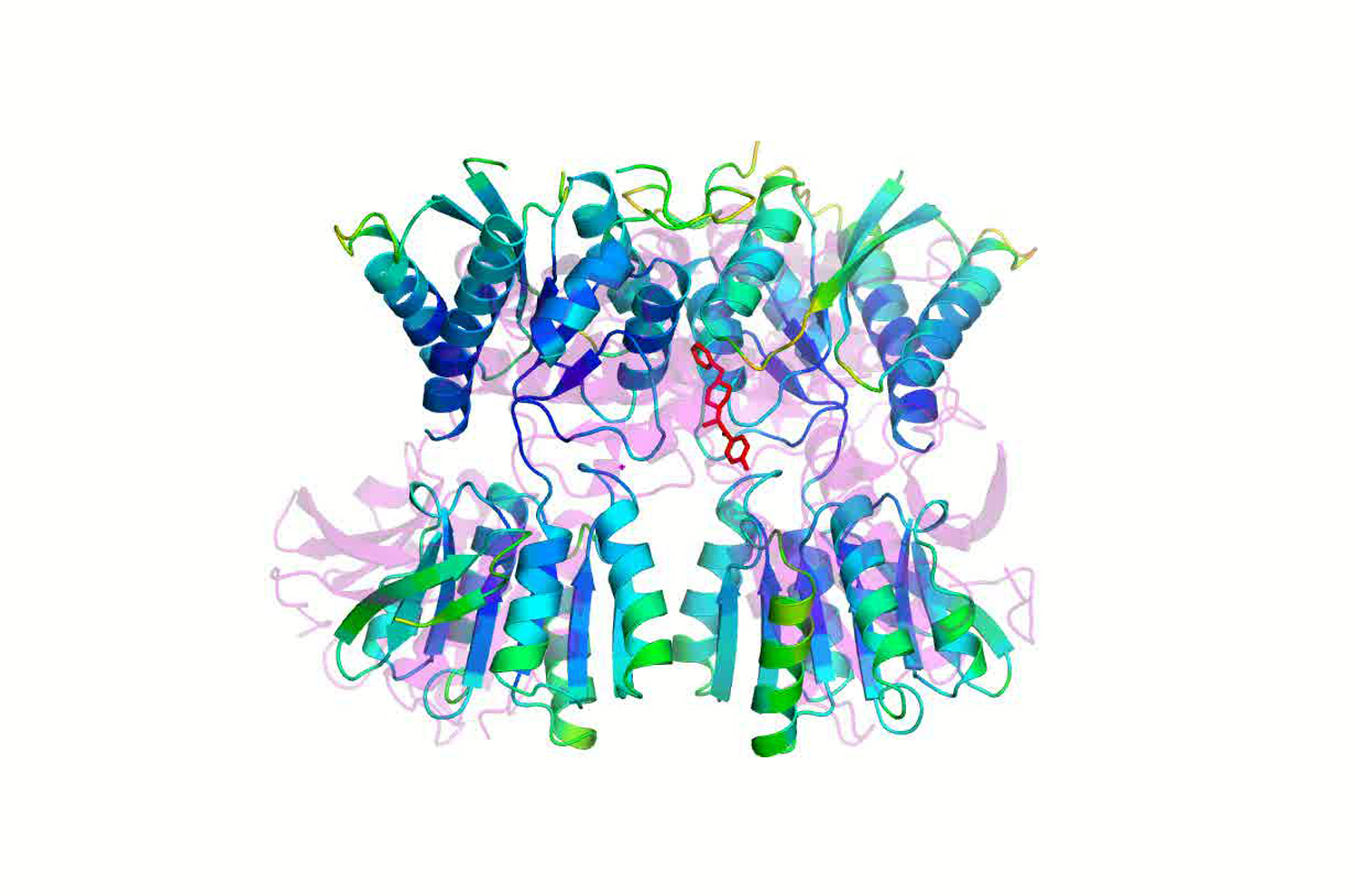

Supplement: Movie S7. Transition from Dimeric Conformer of AMPA GluA3 to Heterodimeric NMDAR NTD, Related to Figure 6 [file mmc8.jpg]
